# MicroRNA-6084 orchestrates angiogenesis and liver metastasis in colorectal cancer via extracellular vesicles

**DOI:** 10.1172/jci.insight.189503

**Published:** 2025-06-10

**Authors:** Yang Zhang, Xuyang Yang, Su Zhang, Qing Huang, Sicheng Liu, Lei Qiu, Mingtian Wei, Xiangbing Deng, Wenjian Meng, Hai-Ning Chen, Yaguang Zhang, Junhong Han, Ziqiang Wang

**Affiliations:** 1 Colorectal Cancer Center,; 2 Department of General Surgery, and; 3 Research Laboratory of Tumor Epigenetics and Genomics, Department of General Surgery, Frontiers Science Center for Disease-related Molecular Network and National Clinical Research Center for Geriatrics, State Key Laboratory of Biotherapy and Cancer Center, West China Hospital, Sichuan University, Chengdu, China.

**Keywords:** Angiogenesis, Gastroenterology, Oncology, Colorectal cancer, Hypoxia, Noncoding RNAs

## Abstract

The prognosis for colorectal cancer (CRC) patients with liver metastasis remains poor, and the molecular mechanisms driving CRC liver metastasis are not fully understood. Tumor-derived hypoxia-induced extracellular vesicles have emerged as key players in inducing angiogenesis by transferring noncoding RNAs. However, the specific role of CRC-derived hypoxic extracellular vesicles (H-EVs) in regulating premetastatic microenvironment (PMN) formation by inducing angiogenesis remains unclear. Our study demonstrates that H-EVs induce angiogenesis and liver metastasis. Through microRNA microarray analysis, we identified a reduction in miR-6084 levels within H-EVs. We found that miR-6084 inhibited angiogenesis by being transferred to endothelial cells via EVs. In endothelial cells, miR-6084 directly targeted angiopoietin like 4 (*ANGPTL4*) mRNA, thereby suppressing angiogenesis through the ANGPTL4-mediated JAK2/STAT3 pathway. Furthermore, we uncovered that specificity protein 1 (SP1) acted as a transcription factor regulating miR-6084 transcription, while hypoxia-inducible factor 1A (HIF1A) decreased miR-6084 expression by promoting SP1 protein dephosphorylation and facilitating ubiquitin-proteasome degradation in SW620 cells. In clinical samples, we observed low expression of miR-6084 in plasma-derived EVs from CRC patients with liver metastasis. In summary, our findings suggest that CRC-derived H-EVs promote angiogenesis and liver metastasis through the HIF1A/SP1/miR-6084/ANGPTL4 axis. Additionally, miR-6084 holds promise as a diagnostic and prognostic biomarker for CRC liver metastasis.

## Abstract 2

MicroRNA-6084 orchestrates angiogenesis and liver metastasis in CRC via EVs. It holds promise as a potential diagnostic and prognostic biomarker for CRC liver metastasis.

## Graphical abstract

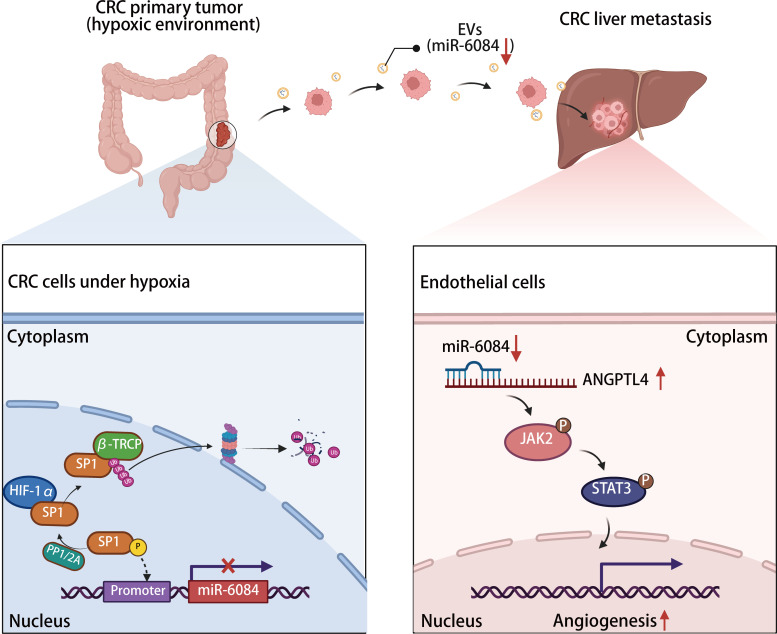

## Introduction

Colorectal cancer (CRC) ranks third in cancer incidence and is the second leading cause of cancer-related death worldwide (1). In recent years, the prognosis of patients with CRC has been greatly improved because of the establishment of total mesorectal excision or complete mesocolic excision and the application of selective neoadjuvant chemoradiotherapy, targeted therapy, immunotherapy, and so on (2–4). However, approximately 30%–50% of patients still experience CRC recurrence or metastasis after curative resection of CRC (5). Liver metastasis is the most common site of distant metastasis in CRC, accounting for 75% of all recurrent and metastatic cases. Approximately 15%–25% of patients with CRC present with simultaneous liver metastasis at initial diagnosis, and most patients with liver metastases are initially ineligible for radical resection (6). Moreover, the 5-year overall survival and recurrence-free survival of patients with complete resection for liver metastases remain poor (7). Therefore, gaining a better understanding of the molecular mechanisms underlying CRC liver metastasis is of great significance and clinical value for accurate diagnosis and treatment of CRC liver metastasis and for improving the prognosis of patients with CRC.

CRC liver metastasis is a multistep biological process encompassing evasion from the primary CRC, migration through the extracellular matrix, invasion into neighboring tissues, survival in circulation, extravasation, and colonization in the distant liver, among others (8). This intricate process involves various complex factors, including the tumor microenvironment (TME), the immune system, and the premetastatic microenvironment (PMN). The PMN was first proposed by David Lyden (9) and was defined as “a supportive and receptive tissue microenvironment undergoing a series of molecular and cellular changes to form the metastatic-designated sites,” characterized by inflammation, angiogenesis and vascular permeability, lymphangiogenesis, immunosuppression, organotropism, and reprogramming. The pivotal components of the PMN include tumor-derived secreted factors, extracellular vesicles (EVs), bone marrow–derived cells, and so on (10). Studies have highlighted the crucial role of PMN in promoting early CRC metastasis (8, 11). Our previous study also found that CRC-derived EVs can induce a liver-immunosuppressive PMN formation to promote tumor early liver metastasis (12).

EVs are small, membrane-bound vesicles released by cells into the extracellular environment (13), carrying a diverse array of bioactive molecules, such as proteins, lipids, and nucleic acids. They play a pivotal role in intercellular communication and regulate various physiological and pathological processes (14). Recent reports highlight the significant role of tumor-derived EVs in the formation of the PMN (15, 16). Hypoxia and angiogenesis are fundamental features of the TME, intricately linked to the onset and progression of solid tumors. Under hypoxic conditions, hypoxia-inducible factors (HIFs) regulate the transcription of numerous genes, stimulating angiogenesis and promoting metabolic reprogramming, immune evasion, and drug resistance (17). Intercellular communication mediated by EVs plays a critical role in hypoxia-driven angiogenesis processes. Hypoxia enhances the secretion of EVs and alters their contents to regulate downstream tumor angiogenesis mechanisms, thereby promoting tumor progression (18, 19).

A previous study found that CRC-derived EV microRNA 25-3p (miR-25-3p) promotes PMN formation by inducing angiogenesis and vascular permeability. Mechanistically, miR-25-3p can regulate the expression of VEGFR2, tight junction protein 1 (ZO-1), occludin, and Claudin5 in endothelial cells through targeting *KLF2* and *KLF4* (20). Hypoxic tumor-derived EVs were reported to induce angiogenesis via transferring noncoding RNAs in lung cancer, esophageal squamous cell carcinoma, and pancreatic cancer (21–23). However, further investigation is needed to elucidate how CRC-derived hypoxic EVs (H-EVs) specifically regulate PMN formation by inducing angiogenesis.

In this study, we established that CRC H-EVs possess the capability to induce both angiogenesis and liver metastasis. Subsequently, our investigation revealed a reduction in miR-6084 levels within H-EVs, demonstrating its inhibitory effect on angiogenesis both in vitro and in vivo. Through further exploration, we identified the direct target of miR-6084 in HUVECs and delineated the downstream pathway influenced by miR-6084, along with the upstream molecules regulating its expression. Moreover, our findings indicate the potential utility of miR-6084 derived from plasma EVs of patients with CRC as a biomarker for predicting liver metastasis.

## Results

### Characterization of CRC cell-derived EVs under normoxic or hypoxic conditions and their uptake by human umbilical vein endothelial cells.

SW620 cells were cultured under normoxic (20% oxygen concentration) or hypoxic (1% oxygen concentration) conditions for 2 days. The levels of HIF1A expression were significantly elevated under hypoxic conditions (Supplemental Figure 1A; supplemental material available online with this article; https://doi.org/10.1172/jci.insight.189503DS1). EVs derived from SW620-conditioned medium were isolated by ultrafiltration plus size-exclusion chromatography (SEC). The morphology of the isolated EVs was observed by transmission electron microscopy (TEM), which revealed typical lipid bilayer particles 30–150 nm in size (Supplemental Figure 1B). Nanoparticle tracking analysis (NTA) further verified that the majority of particles were approximately 100 nm in size (Supplemental Figure 1C). Western blot analysis demonstrated high expression of EV markers CD9 and TSG101 in both SW620 cells and isolated EVs, while negative controls GRP94 and Calnexin were minimally detected (Supplemental Figure 1D). Collectively, these findings confirm the successful isolation of EVs under both normoxic and hypoxic conditions, meeting the quality standards required for subsequent experiments (24).

To investigate the interactions between normoxic or hypoxic SW620-derived EVs and HUVECs, HUVECs were incubated with DID-labeled EVs. Then, the HUVECs were stained with DAPI and phalloidin. Cellular uptake was assessed using confocal microscopy at 1, 3, and 6 hours postincubation. The results revealed that EVs were gradually absorbed by HUVECs over time (Supplemental Figure 1E). Morphologically, HUVECs treated by H-EVs aggregated into small clusters, which subsequently extend protrusions to interconnect with adjacent clusters, ultimately forming capillary-like tubular structures. The absorbed EVs were predominantly localized in the cytoplasm of HUVECs. In contrast, DID dye alone (PBS control) was not internalized by HUVECs. These results indicated that normoxic and hypoxic SW620-derived EVs can be efficiently absorbed by human umbilical vein endothelial cells (HUVECs) within several hours.

### CRC-derived H-EVs promote HUVECs’ proliferation, migration, and angiogenesis in vitro.

**Figure 1:**
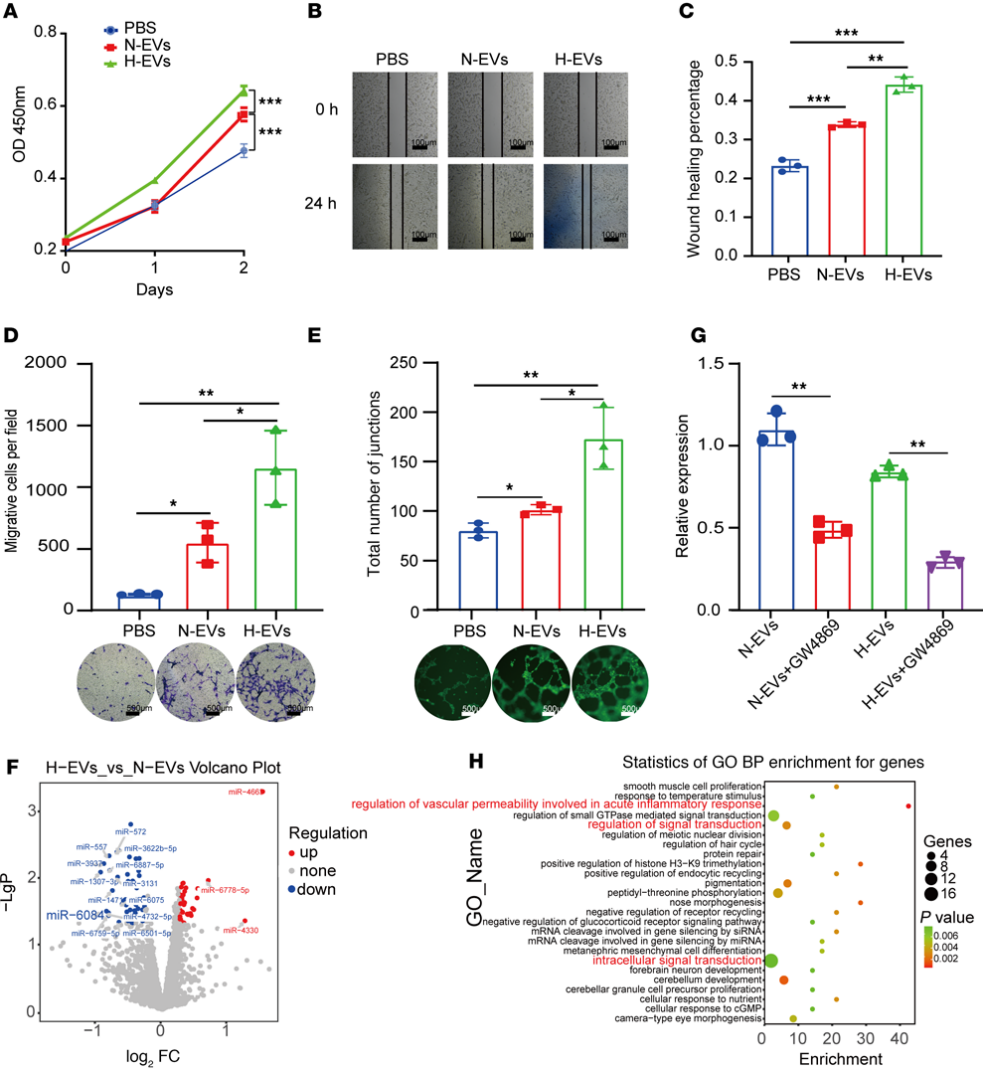
The impact of PBS, N-EVs, and H-EVs on the proliferation, migration, and tube formation of HUVECs and selection of differentially expressed miRNAs between N-EVs and H-EVs. (**A**) The proliferation ability of HUVECs treated with PBS, N-EVs, and H-EVs was analyzed by CCK8 assay. The migration ability was assessed by wound healing assay (**B**) and Transwell assay (**D**). The relative wound healing (%) was calculated (**C**). The number of migrative cells was counted and graphed (**D**). Representative pictures of tube formation were taken after staining with Calcein-AM and quantified by measuring the total vessel length (**E**). Volcano maps visually display differentially expressed genes (|FC| > 1.5) (**F**). SW620 cells were treated with EV secretion inhibitor GW4869, and the expression of miR-6084 was validated using real-time quantitative polymerase chain reaction (RT-qPCR). U6 was used as internal control (**G**). GO analysis suggests that the function of miR-6084 is enriched in regulating vascular permeability and intercellular communication (**H**). Each experiment was conducted at least 3 times. Data were presented as mean ± standard deviation (SD). Statistical significance was assessed with 1-way ANOVA with Tukey’s multiple-comparison test (**A**, **C**–**E**, and **G**), * *P* < 0.05, ** *P* < 0.01, *** *P* < 0.001.

We next investigated the impact of normoxic EVs (N-EVs) and H-EVs on the proliferation, migration, and angiogenesis of HUVECs. Cell counting kit-8 (CCK8) assays revealed that H-EVs significantly promoted the proliferation of HUVECs compared with the N-EVs or PBS (Figure 1A). Wound healing and Transwell assays indicated that H-EVs increased the migration capacity of HUVECs in contrast with the N-EV and PBS groups (Figure 1, B–D). Tube formation assays further indicated that H-EVs induced robust capillary-like structure formation in HUVECs (Figure 1E). We further verified this result using EVs isolated from another CRC cell line, HCT116 (Supplemental Figure 2, A–E). Collectively, these findings suggest that CRC-derived H-EVs promote HUVECs’ proliferation, migration, and angiogenesis in vitro.

### MiR-6084 is the primary negative regulatory molecule in hypoxic SW620-derived EVs.

Our previous systematic review highlighted that the main function of EVs is containing miRNAs to target the recipient cells and alter cellular functions (16). To decipher the pivotal miRNAs orchestrating angiogenesis in hypoxic CRC-derived EVs, we extracted total RNA from H-EVs and N-EVs, then performed expression profiling using an Affymetrix miRNA 4.0 microarray. We identified 40 significantly upregulated miRNAs and 51 downregulated miRNAs (*P* < 0.05, |fold-change [FC]| > 1.2). Upon adjusting to |FC| > 1.5, 3 miRNAs were significantly upregulated, and 13 miRNAs were downregulated, as depicted in the heatmap (Supplemental Figure 3A) and volcano plot (Figure 1F). The detailed information of these 16 differentially expressed miRNAs is described in Supplemental Table 1.

Next, we validated the expression of the differentially expressed miRNAs in SW620 cells and their derived EVs using RT-qPCR. Our data verified the significant upregulation of miR-4663 and the significant downregulation of miR-6084, miR-6759-5p, and miR-6887-5p. MiR-6084 also exhibited consistent repeatability compared with other miRNAs (Supplemental Figure 3B). This finding was further corroborated in SW480 cells and their derived EVs (Supplemental Figure 3C). Given the lack of prior reports on miR-6084, we selected it for further investigation. To confirm whether miR-6084 is encapsulated within EVs, we treated SW620 cells with the EV-secreting inhibitor GW4869. RT-qPCR analysis revealed reduced miR-6084 levels in GW4869-treated cells compared with controls (Figure 1G). Gene Ontology (GO) biological process (BP) analysis revealed that miR-6084–regulated genes were enriched in intracellular signal transduction and regulation of vascular permeability (Figure 1H).

Collectively, these results underscore the reduction of miR-6084 in CRC-derived H-EVs and its predominant encapsulation within EVs.

### MiR-6084 inhibits HUVECs’ proliferation, migration, and angiogenesis in vitro.

**Figure 2:**
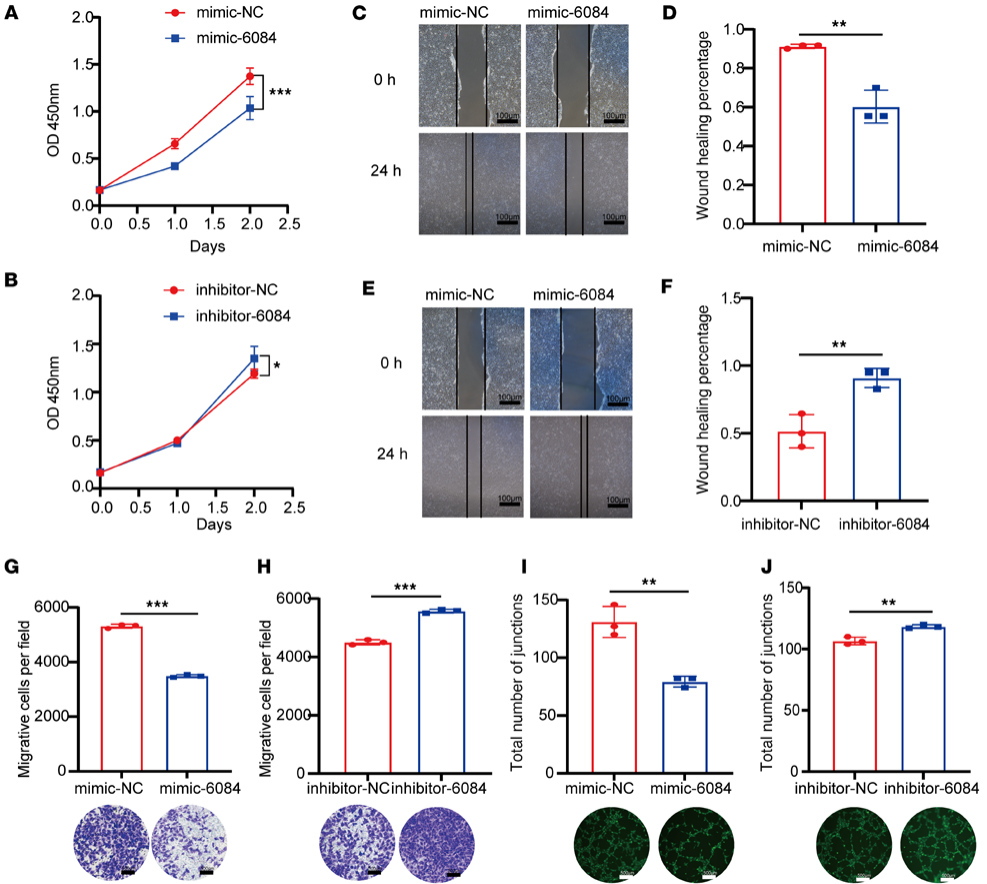
MiR-6084 inhibits angiogenesis in vitro. (**A** and **B**) The proliferation ability of HUVECs was assessed by CCK8 assay. NC, normal control. (**C** and **E**) The migration ability was assessed by wound healing assay. (**D** and **F**) The relative wound healing (%) was calculated. (**G** and **H**) The migration ability was assessed by Transwell assay. The number of migrative cells was counted and graphed. Scale bar, 500 μm. (**I** and **J**) Representative pictures of tube formation were taken after staining with Calcein-AM. The tube formation ability was quantified by measuring the total number of junctions. Scale bar, 500 μm. Each experiment was conducted at least 3 times. Data were presented as mean ± SD. Statistical significance was assessed with 2-tailed unpaired Student’s *t* test (**A**, **B**, **D**, **F**–**J**), * *P* < 0.05, ** *P* <0.01, *** *P* < 0.001.

To further explore the function of miR-6084, we examined the impact of the miR-6084 mimic and inhibitor on the proliferation, migration, and angiogenesis of HUVECs. CCK8 assays revealed that the miR-6084 mimic inhibited the proliferation of HUVECs, while the miR-6084 inhibitor enhanced it (Figure 2, A and B). Wound healing and Transwell assays demonstrated that the miR-6084 mimic reduced HUVEC migration, whereas the inhibitor promoted it (Figure 2, C–H). Furthermore, tube formation assays showed that the miR-6084 inhibitor could induce capillary-like structure formation, while the mimic inhibited this process (Figure 2, I and J). Overall, these results suggest that miR-6084 inhibits HUVECs’ proliferation, migration, and angiogenesis in vitro.

### MiR-6084 functions as an angiogenesis inhibitor by directly targeting angiopoietin-like protein 4.

**Figure 3:**
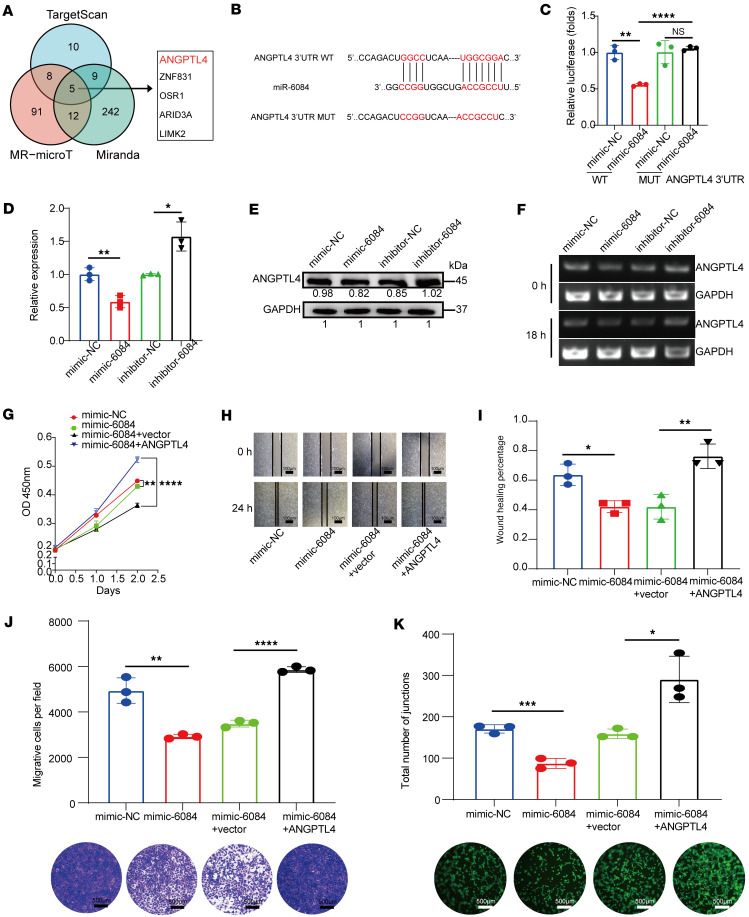
MiR-6084 inhibits angiogenesis by directly targeting ANGPTL4. (**A**) Three databases were applied to predict the potential targets of miR-6084. (**B**) The predicted binding sites of ANGPTL4 and miR-6084. WT, wild-type; MUT, mutant. (**C**) Luciferase activities of ANGPTL4 WT or MUT 3′UTR in HUVECs after transfection of miR-31-5p mimic. (**D**) Expression level of *ANGPTL4* after transfection of miR-6084 mimic or inhibitor by RT-qPCR. β-Actin was used as internal control. (**E**) Expression of ANGPTL4 after transfection of miR-6084 mimic or inhibitor by Western blot. (**F**) The stability of *ANGPTL4* mRNA was analyzed by an actinomycin D treatment experiment and agarose gel nucleic acid electrophoresis. (**G**) The proliferation ability of HUVECs was assessed by CCK8 assay. (**H** and **I**) The relative migration distance of wound healing was calculated. (**J**) The migration ability was assessed by Transwell assay, and the number of migrative cells were counted. (**K**) Representative pictures of tube formation were taken after staining with Calcein-AM. The tube formation ability was quantified by measuring the total number of junctions. Each experiment was conducted at least 3 times. Data were presented as mean ± SD. Statistical significance was assessed with 1-way ANOVA with Tukey’s multiple-comparison test (**C**, **D**, **G**, and **I**–**K**), * *P* < 0.05, ** *P* < 0.01, **** *P* < 0.0001.

MiRNAs typically exert various biological functions mainly by binding to complementary target mRNAs, leading to either translational inhibition or mRNA degradation (25). Using TargetScan, Miranda, and MR-microT databases, we identified 5 potential miR-6084 target mRNAs: angiopoietin like 4 (*ANGPTL4*), *ZNF831*, *OSR1*, *ARID3A*, and *LIMK2* (Figure 3A). Given the association of ANGPTL4 with angiogenesis, we selected it for further investigation. Figure 3B shows the predicted binding site between miR-6084 and the 3′ untranslated region (UTR) of *ANGPTL4* mRNA. Dual-luciferase reporter assays revealed that miR-6084 mimics reduced luciferase activity in WT ANGPTL4-transfected HUVECs but not in MUT *ANGPTL4*-transfected cells, validating *ANGPTL4* as a direct target of miR-6084 (Figure 3C). We further investigated whether this binding leads to translational inhibition or *ANGPTL4* mRNA degradation. RT-qPCR and Western blotting demonstrated that the miR-6084 mimic or inhibitor could alter both the mRNA and protein levels of ANGPTL4 (Figure 3, D and E), indicating miR-6084 mediated *ANGPTL4* mRNA degradation. Actinomycin D treatment further validated reduced *ANGPTL4* mRNA stability in the presence of miR-6084 mimics (Figure 3F).

Rescue assays using an *ANGPTL4*-overexpression plasmid reversed the effects of miR-6084 mimic on HUVEC proliferation, migration, and angiogenesis (Figure 3, G–K). In conclusion, these findings affirm that *ANGPTL4* is the direct target of miR-6084, with their binding resulting in *ANGPTL4* mRNA degradation. MiR-6084 exerts inhibitory effects on proliferation, migration, and angiogenesis by regulating *ANGPTL4*.

### MiR-6084 suppresses angiogenesis through the ANGPTL4-mediated JAK2/STAT3 pathway.

Kyoto Encyclopedia of Genes and Genomes pathway enrichment analysis revealed that miR-6084–targeted genes were enriched in the Ras/Raf/ERK/MAPK pathway (Supplemental Figure 4A). Moreover, the PI3K/AKT, JAK2/STAT3, and Wnt/β-catenin pathways were reported to be involved in ANGPTL4-mediated angiogenesis (26–28). Western blotting demonstrated significant alterations in STAT3/phosphorylated STAT3 (p-STAT3) levels (Supplemental Figure 4B). Subsequently, rescue assays verified that *ANGPTL4* overexpression reversed miR-6084–induced JAK2/STAT3 pathway downregulation (Supplemental Figure 4C). The protein-protein interaction network constructed using STRING revealed the potential interaction between ANGPTL4 and STAT3 (Supplemental Figure 4D). The correlation of ANGPTL4 and STAT3 expression was further assessed via GEPIA2, showing a positive correlation (*R* = 0.37; Supplemental Figure 4E). Furthermore, the direct interaction between STAT3 and ANGPTL4 was validated using an immunoprecipitation (IP) assay (Supplemental Figure 4F). In summary, our results indicate that miR-6084 inhibits angiogenesis through the ANGPTL4-mediated JAK2/STAT3 pathway.

### HIF1A decreases miR-6084 expression by regulating specificity protein 1 protein ubiquitination and proteasomal degradation.

**Figure 4:**
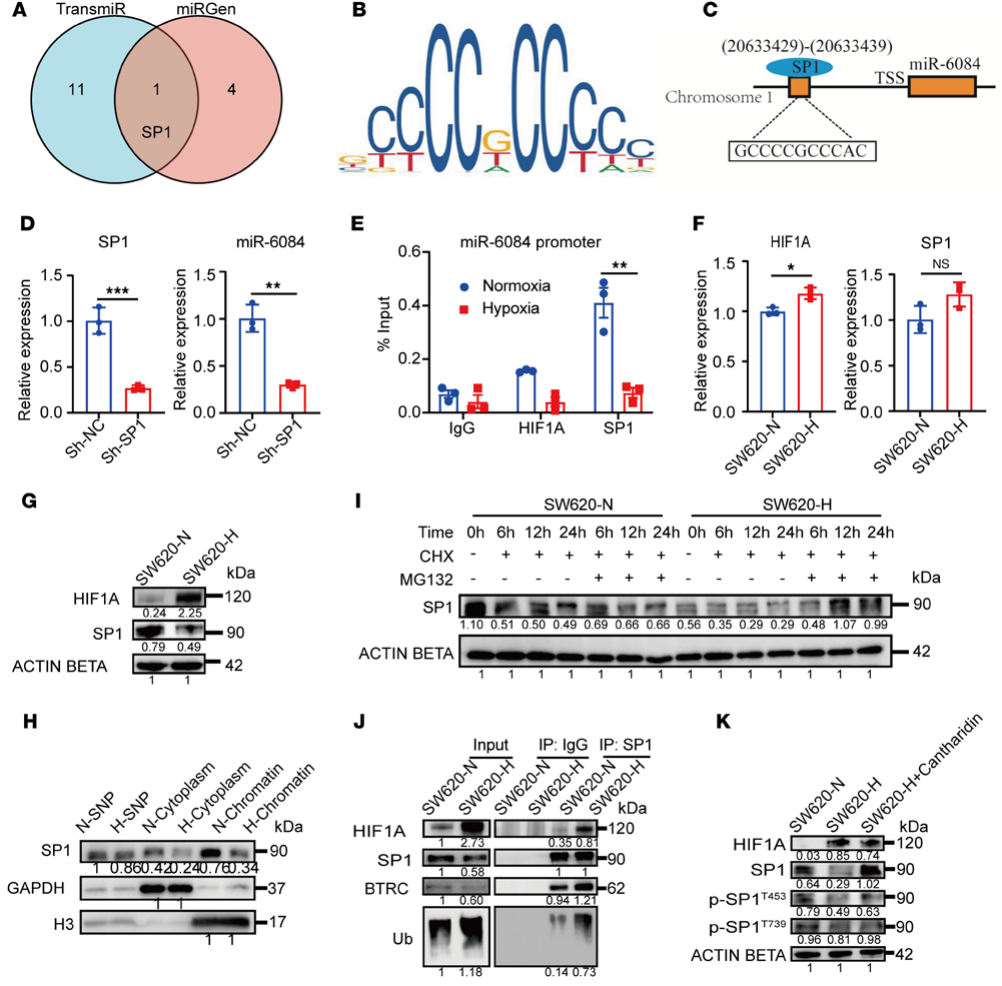
HIF1A decreases miR-6084 expression by regulating SP1 protein ubiquitination and proteasomal degradation. (**A**) Two databases were applied to predict the potential transcription factors of miR-6084. (**B**) The DNA target sequence logos of transcription factor SP1 predicted by JASPAR database. (**C**) A schematic diagram of SP1-binding sites in the promoter of miR-6084. (**D**) Expression level of *SP1* mRNA was detected by RT-qPCR after knockdown of SP1. β-Actin was used as internal control. Expression level of miR-6084 detected by RT-qPCR after knockdown of SP1. U6 was used as internal control. Sh, short hairpin RNA. (**E**) ChIP assays were performed to verify the binding between SP1, HIF1A, and the miR-6084 promoter in SW620. (**F**) Expression level of *HIF1A* mRNA in SW620 under normoxic or hypoxic condition detected by RT-qPCR. Expression level of *SP1* mRNA in SW620 under normoxic or hypoxic condition detected by RT-qPCR. β-Actin was used as internal control. (**G**) Expression level of HIF1A and SP1 in SW620 under normoxic (-N) or hypoxic (-H) condition detected by Western blot. (**H**) Nucleocytoplasmic separation was performed and expression level of SP1 in SW620 nucleus and cytoplasm under normoxic or hypoxic condition was detected by Western blot. SNP, soluble nuclear protein; H3, histone H3. (**I**) SW620 cells were treated by CHX (protein synthesis inhibitor) and MG132 (proteasome inhibitor), and SP1 protein stability was measured by Western blot. (**J**) IP assays were conducted to verify the binding between SP1 and HIF1A, BTRC, or Ub. (**K**) Expression level of p-SP1 in SW620 under normoxic/hypoxic condition or treated by protein phosphatase 1/2A (PP1/2A) cantharidin detected by Western blot. Each experiment was conducted at least 3 times. Data were presented as mean ± SD. Statistical significance was assessed with 2-tailed unpaired Student’s *t* test (**D**–**F**), * *P* < 0.05, ** *P* < 0.01, *** *P* < 0.001.

To investigate the upstream regulatory factors governing miR-6084, we predicted transcription factors influencing the miR-6084 promoter utilizing the TransmiR and miRGen databases. Notably, specificity protein 1 (SP1) emerged as the sole transcription factor predicted by both databases (Figure 4A). Figure 4B shows the DNA target sequence logos of SP1 predicted by the JASPAR database. Furthermore, we generated a schematic diagram illustrating the binding sites of the transcription factor SP1 within the miR-6084 promoter, as predicted via the JASPAR database (Figure 4C). Subsequently, SP1 knockdown reduced miR-6084 levels (Figure 4D). A chromatin immunoprecipitation (ChIP) assay was performed to further verify whether SP1 transcriptionally regulated miR-6084 expression. The results showed that SP1 could bind to the miR-6084 promoter, with a significant reduction observed in SP1 binding under hypoxic conditions (Figure 4E). These outcomes validate SP1 as a transcription factor governing the transcription of miR-6084.

Next, we explored the relationship between SP1 and HIF1A. The results revealed hypoxia decreased SP1 protein levels without affecting mRNA expression (Figure 4, F and G). In addition, nucleocytoplasmic separation revealed reduced chromatin-associated SP1 under hypoxia (Figure 4H). The regulatory interaction between SP1 and HIF1A was also verified in the HCT116 cell line (Supplemental Figure 5, A–D). Collectively, these results indicated that HIF1A regulated the level of SP1 via posttranscriptional mechanisms.

Subsequently, we further explored whether HIF1A affects the degradation of SP1. SW620 cells were treated with cycloheximide (CHX), a protein synthesis inhibitor. Under hypoxic conditions, SP1 exhibited accelerated degradation (Supplemental Figure 6, A and B). Protein degradation primarily occurs via the autophagy/lysosome pathway and the ubiquitin (Ub)/proteasome pathway, both of which play pivotal roles in maintaining cellular homeostasis (28). To discern which pathway was involved in hypoxia-induced degradation of the SP1 protein, SW620 cells were treated with CHX plus MG132, a proteasome inhibitor, or CHX plus chloroquine, an autophagy inhibitor, followed by assessment of SP1 protein stability via Western blotting. While MG132 significantly attenuated hypoxia-induced degradation of the SP1 protein, chloroquine had no effect on this process (Figure 4I and Supplemental Figure 6C). Hence, SP1 degradation primarily occurs via the Ub/proteasome pathway. E3 Ub ligase determines the specific recognition of target proteins and plays an important role in the Ub/proteasome pathway. Thus, we further explored the E3 Ub ligase for SP1 degradation. UbiBrowser, an integrated bioinformatics platform, provides information on known and predicted Ub ligase/deubiquitinase-substrate interactions (29). The results showed that beta-transducin repeat containing E3 ubiquitin protein (BTRC) and RNF4 are E3 ligases (Supplemental Figure 6D), as previously reported (30, 31). IP assays validated the binding between E3 ligases and SP1. Under hypoxic conditions, SP1 exhibited increased binding to BTRC and Ub (Figure 4J). In summary, our findings elucidate that HIF1A decreases miR-6084 expression through BTRC-mediated Ub-proteasome degradation of SP1.

### HIF1A decreases miR-6084 expression by sequestering SP1 from the miR-6084 promoter.

It was reported that HIF1A can suppress the transcription of PKA regulatory subunit 2B (PRKAR2B) by sequestering SP1 from the PRKAR2B promoter in human growth hormone–secreting pituitary tumors (32). We sought to investigate whether a similar mechanism occurs with miR-6084 transcription regulation by HIF1A in CRC. Initially, immunofluorescence was conducted to validate the colocalization of SP1 and HIF1A (Supplemental Figure 7A). Co-IP assays were subsequently performed to further verify the interaction between SP1 and HIF1A (Supplemental Figure 7, B and C). Under hypoxic conditions, HIF1A accumulation leads to increased binding of HIF1A to SP1, resulting in the displacement of SP1 from the miR-6084 promoter. Ultimately, this process leads to decreased transcription of miR-6084.

### Hypoxia promotes the dephosphorylation of SP1 at Thr739 and Thr453 via phosphatase 2A.

It has been reported that phosphorylation of SP1 at Thr739 or Thr453 can confer protection against Ub-dependent degradation (33, 34), with protein phosphatase 2A (PP2A) implicated in mediating the dephosphorylation of SP1 (35). To investigate this mechanism, SW620 cells were cultured under normoxic or hypoxic conditions or treated with cantharidin, a PP2A inhibitor. Hypoxia downregulates the expression of SP1, p-SP1 (Thr739), and p-SP1 (Thr453). However, this effect was reversed by cantharidin treatment (Figure 4K). These results indicated that hypoxia promotes the dephosphorylation of SP1 at Thr739 and Thr453 via PP2A, consequently facilitating Ub-proteasome degradation of SP1.

### CRC-derived H-EVs promote angiogenesis and liver metastasis in vivo.

To assess the impact of CRC-derived H-EVs on angiogenesis and liver metastasis in vivo, we initially conducted EV uptake assays to determine their organ distribution in male mice. DID dye–labeled EVs (DID^+^EVs) or DID dye was injected via the tail vein for 30 minutes or 24 hours. In vivo imaging revealed predominant accumulation of EVs in the liver and gastrointestinal tract, with prolonged retention compared with DID dye (Supplemental Figure 8A). Then, the mice were sacrificed, and the liver, lungs, heart, spleen, kidneys, gastrointestinal tract, and femurs were excised for ex vivo imaging. The results verified that EVs were primarily distributed in the liver and gastrointestinal tract (Supplemental Figure 8B).

**Figure 5:**
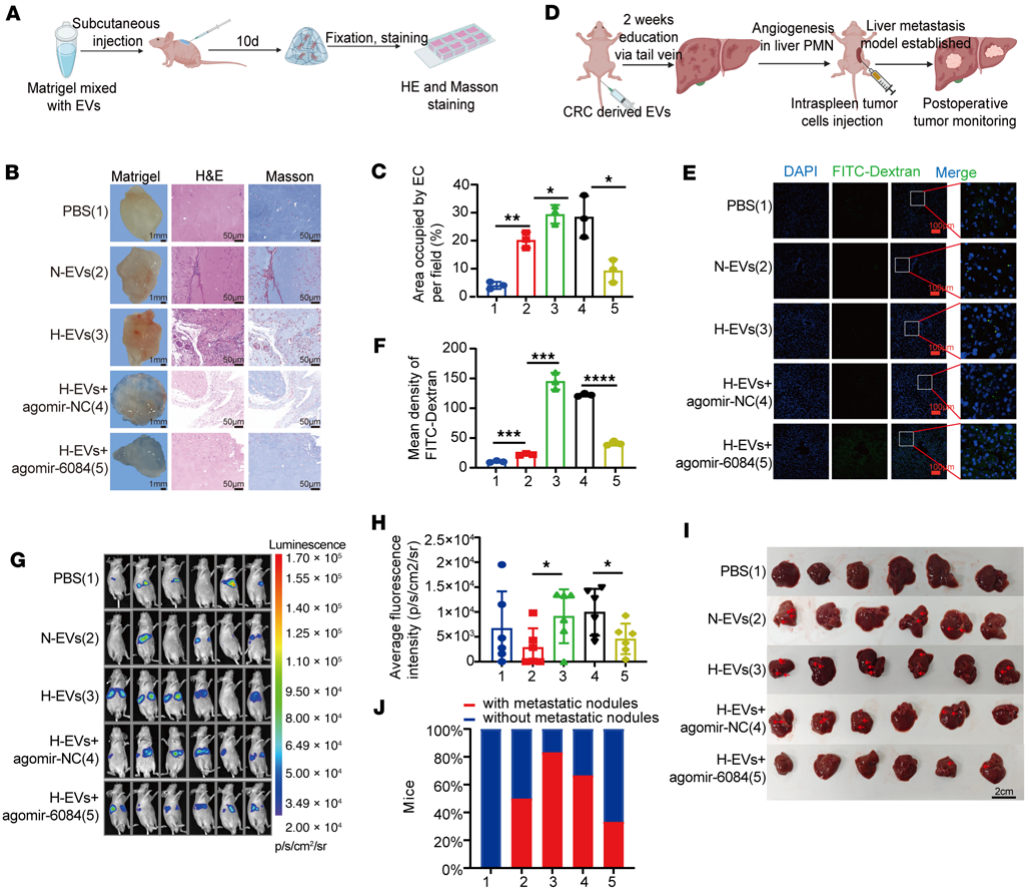
CRC-derived H-EVs promote angiogenesis and liver metastasis through EV miR-6084 in vivo. (**A**) The flowchart of Matrigel plug-in assays. (**B**) Representative images of the Matrigel plugs. H&E and Masson’s staining of the Matrigel plug. (**C**) Area occupied by endothelial cells (EC) per field was measured to assess neovascularization. (**D**) The flowchart of liver metastasis assays. (**E**) FITC-Dextran was injected into mice through the tail vein 30 minutes before sacrifice. Liver was removed for immunofluorescence to capture FITC flowing from microvessels. (**F**) Mean FITC-Dextran per field was measured to assess neovascularization. (**G**) Representative images of liver metastasis in nude mice after splenic injection of SW620 cells. (**H**) The fluorescence intensity of each mouse (p/s/cm^2^/sr). (**I**) Liver images showed more metastatic nodules on the liver surface. (**J**) Quantifying the percentage of mice with metastatic nodules. Each experiment was conducted at least 3 times. Data were presented as mean ± SD. Statistical significance was assessed with 1-way ANOVA with Tukey’s multiple-comparison test (**C**, **F**, and **H**), * *P* < 0.05, ** *P* < 0.01, *** *P* < 0.001, **** *P* < 0.0001.

Next, Matrigel plug-in assays were performed to explore the angiogenic potential of CRC-derived H-EVs in vivo (Figure 5A). As expected, the Matrigel plugs containing H-EVs exhibited enhanced vascularization compared with those containing N-EVs. However, Matrigel plugs containing H-EVs and miR-6084 agomir showed reduced vascularization compared with those containing H-EVs and agomir-NC (Figure 5, B and C). Subsequently, liver premetastasis and metastasis models were established (Figure 5D). Mice were pretreated with SW620-derived EVs for 2 weeks to remodel the PMN. Following this, FITC-Dextran was injected via the tail veins to validate liver vascular permeability. Immunofluorescence imaging revealed that H-EV increased liver vascular permeability, whereas miR-6084 mitigated this effect (Figure 5, E and F).

Additionally, after liver PMN formation, intrasplenic injection of SW620 cells established liver metastatic models. In vivo imaging demonstrated that H-EVs promoted liver metastasis, while miR-6084 inhibited liver metastasis (Figure 5, G–J). Furthermore, CD31 immunohistochemistry (IHC) revealed that H-EV increased liver neovascular density, while miR-6084 attenuated neovascular density after CRC liver metastasis (Supplemental Figure 9, A and B).

Taken together, these results revealed that CRC-derived H-EVs were primarily distributed in the liver and promoted angiogenesis and liver metastasis in vivo.

### MiR-6084 is a potential diagnostic and prognostic biomarker for liver metastasis in patients with CRC.

**Figure 6:**
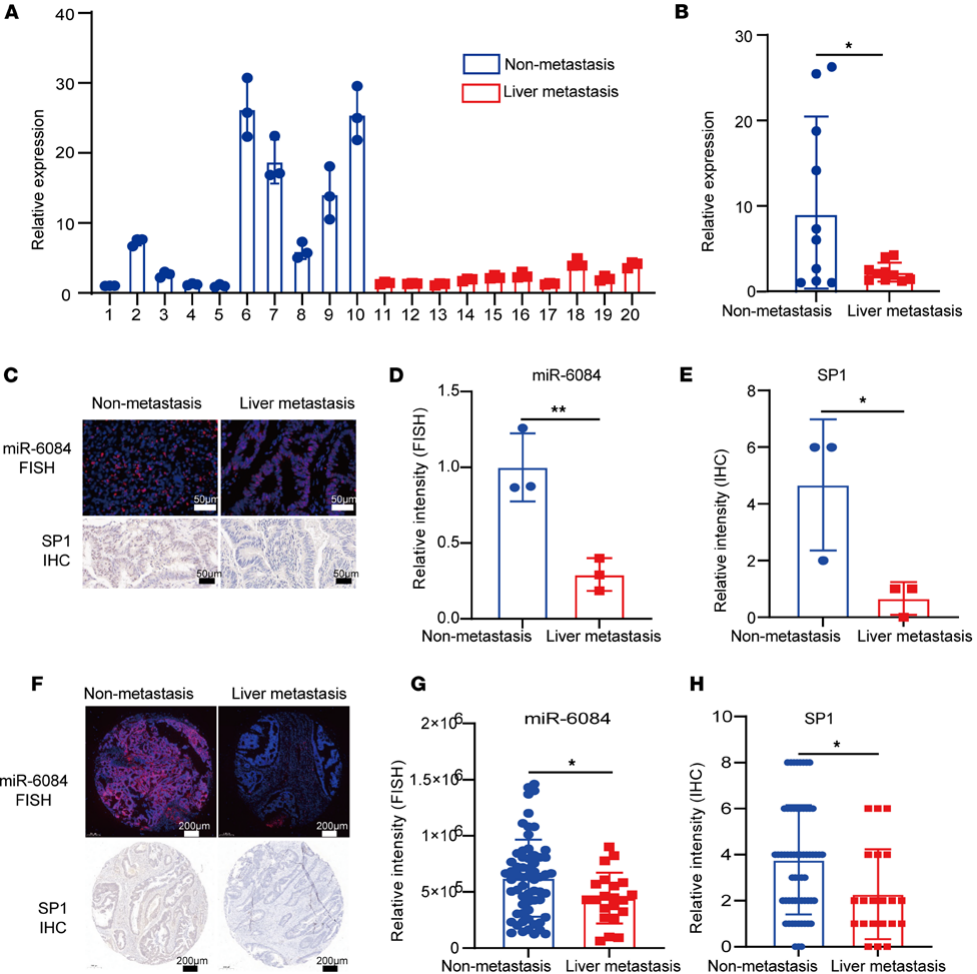
MiR-6084 is correlated with liver metastasis of patients with CRC. (**A**) Levels of miR-6084 in the plasma-derived EVs of CRC patients with or without liver metastasis detected by RT-qPCR. (**B**) Unpaired 2-tailed Student’s *t* test was performed between nonmetastasis group and liver metastasis group. (**C**) Representative RNA-FISH images of miR-6084 expression and representative IHC image of SP1 in CRC samples obtained from our hospital. (**D** and **E**) The relative intensity of miR-6084 and SP1 was compared. (**F**) Representative RNA-FISH images of miR-6084 expression and representative IHC image of SP1 in CRC tissue chips. (**G** and **H**) The relative intensity of miR-6084 and SP1 in CRC tissue chips was compared. Each experiment was conducted at least 3 times. Data were presented as mean ± SD. Statistical significance was assessed with 2-tailed unpaired Student’s *t* test (**B**, **D**, **E**, **G**, and **H**), * *P* < 0.05, ** *P* < 0.01.

To assess the potential of miR-6084 as a diagnostic biomarker for CRC liver metastasis, we collected blood samples from CRC patients with or without liver metastasis. Then, we quantified the levels of miR-6084 in plasma using RT-qPCR. The results showed that the levels of miR-6084 were comparable between the 2 groups (Supplemental Figure 10, A and B). Next, we isolated EVs from plasma and characterized them by TEM, NTA, and Western blot (Supplemental Figure 11, A–C). Similarly, we quantified the levels of miR-6084 in the plasma-derived EVs using RT-qPCR. The results demonstrated that miR-6084 exhibited lower expression levels in the plasma-derived EVs of CRC patients with liver metastasis (Figure 6, A and B).

Additionally, we obtained 6 primary CRC tissues from our hospital and performed miR-6084 fluorescence in situ hybridization (FISH) and SP1 IHC. The results revealed reduced expression levels of miR-6084 and SP1 in primary tumor tissues from patients with metastatic CRC (Figure 6, C–E). Further validation utilizing a tissue microarray containing samples from 83 patients (21 with liver metastasis) verified these findings (Supplemental Figure 12A). Consistently, miR-6084 and SP1 exhibited low expression in CRC tissues from patients with liver metastases (Figure 6, F–H, and Supplemental Figure 12, C and D). Moreover, the expression levels of miR-6084 and SP1 showed trends toward correlation with patient survival (Supplemental Figure 12, F–I). In summary, these results demonstrated the reduced expression level of miR-6084 in plasma-derived EVs and CRC tissues with CRC liver metastasis. MiR-6084 has the potential to serve as a diagnostic and prognostic biomarker for CRC liver metastasis.

## Discussion

**Figure 7:**
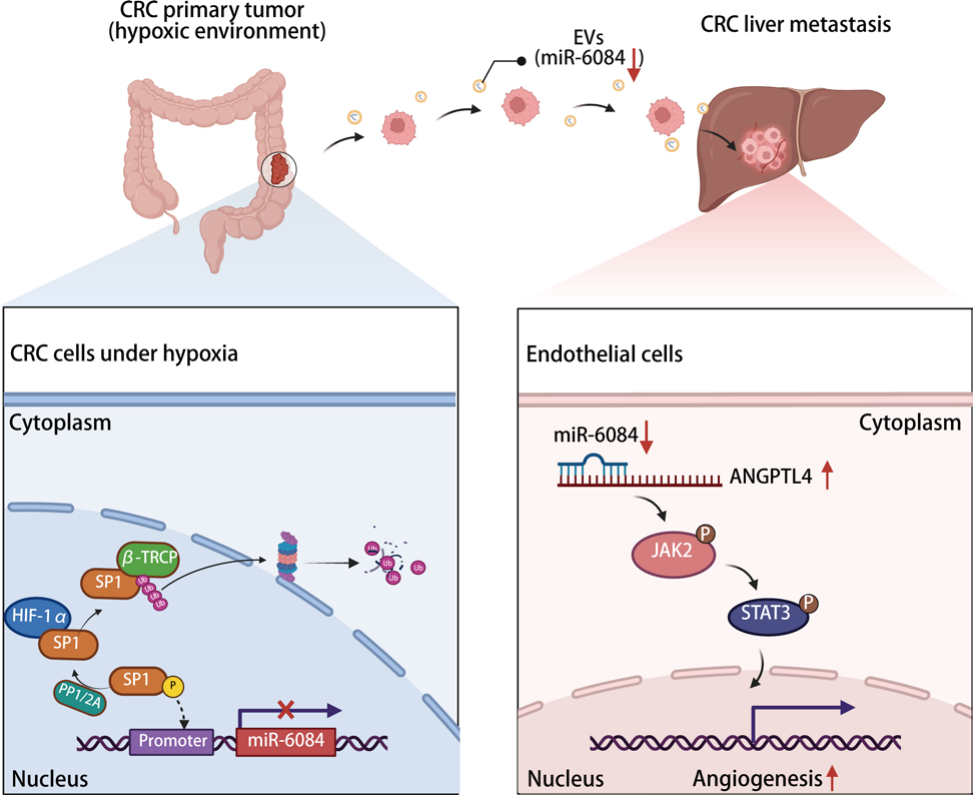
The proposed model of CRC-derived H-EVs promoting angiogenesis and liver metastasis through the HIF1A/SP1/miR-6084/ANGPTL4 axis. H-EVs induce both angiogenesis and liver metastasis, and the level of miR-6084 within H-EVs is reduced. MiR-6084 inhibits angiogenesis by transferring to endothelial cells via EVs. In endothelial cells, miR-6084 directly targets *ANGPTL4* mRNA, thus inhibiting angiogenesis through the ANGPTL4-mediated JAK2/STAT3 pathway. Moreover, SP1 acts as a transcription factor regulating miR-6084 transcription, while HIF1A promotes the dephosphorylation of SP1 at Thr739 and Thr453 via PP2A, consequently facilitating Ub-proteasome degradation of SP1.

Liver metastasis represents the most prevalent form of distant metastasis in CRC (6). Notably, liver metastasis is not confined to advanced-stage patients; even early-stage CRC cases can develop liver metastasis, which significantly worsens patient prognosis (36). Given its clinical significance, understanding the underlying molecular mechanisms of CRC liver metastasis is crucial for developing effective therapeutic strategies. In the current study, we demonstrated the capacity of CRC H-EVs to stimulate both angiogenesis and liver metastasis. Subsequently, we identified a reduction in miR-6084 levels within H-EVs, unveiling its role in inhibiting angiogenesis via transfer to HUVECs through EVs. Furthermore, we identified *ANGPTL4* as the direct target of miR-6084 in HUVECs, revealing the miRNA’s ability to impede angiogenesis by modulating the JAK2/STAT3 pathway through ANGPTL4. To further understand the regulatory network, we delved into the transcriptional mechanism governing miR-6084 expression and identified SP1 as a pivotal transcription factor orchestrating miR-6084 transcription. Notably, we delineated how HIF1A regulates miR-6084 expression by controlling SP1 protein ubiquitination and proteasomal degradation in SW620 cells (Figure 7). Conclusively, our clinical data underscored the potential of miR-6084 derived from plasma EVs of patients with CRC as a promising biomarker for liver metastasis onset.

Paget’s influential “seed and soil” hypothesis has profoundly shaped our understanding of tumor metastasis, illustrating that the successful colonization of metastatic cancer cells depends on the compatibility between the characteristics of the soil (target organs) and the seeds (cancer cells) (37). Building upon this foundation, the concept of the PMN, as an extension of Paget’s theory, has emerged as a fundamental framework for exploring the mechanisms governing tumor distant metastasis (9, 38). Accumulating studies have highlighted the pivotal role of tumor-derived EVs in inducing PMN formation, thereby driving tumor progression and metastasis (15, 16). In the context of CRC, EVs derived from these tumors have been implicated in inducing PMN formation and promoting CRC liver metastasis by polarizing M2 macrophages. Mechanistically, EV-mediated delivery of miR-934 downregulates PTEN expression, consequently activating the PI3K/AKT signaling pathway within macrophages (39). Additionally, emerging investigations have shown that CRC-derived EVs carrying miR-181a-5p can trigger the activation of hepatic stellate cells (HSCs) into cancer-associated fibroblasts (CAFs) by targeting SOCS3 and stimulating the IL-6/STAT3 pathway. These activated HSCs, in turn, secrete the chemokine CCL20, which activates the ERK1/2 pathway, thereby reshaping the TME and facilitating liver metastasis (40). The PMN is further characterized by both an inflammatory response and immunosuppression. One study has uncovered that CRC-derived EVs containing miR-21 play a crucial role in establishing a proinflammatory liver phenotype, thereby promoting liver metastasis by binding to Toll-like receptor 7 (41). Moreover, our previous research has established that CRC-derived EV TGFB1 induces the formation of an immunosuppressive PMN in the liver, which facilitates early liver metastasis by recruiting myeloid-derived suppressor cells that inhibit NK cell cytotoxicity (12).

Beyond the pivotal roles of M2 macrophage polarization, activation of CAFs, inflammatory responses, and immunosuppression previously discussed, angiogenesis emerges as a crucial factor in PMN formation and tumor metastasis (42, 43). Tumors cannot grow beyond 2–3 mm in size without neovascularization, which provides essential oxygen and nutrients for their sustenance (44). Mounting evidence indicates that tumor-derived EVs profoundly affect angiogenesis and vascular permeability by transporting various pro-angiogenic or antiangiogenic biomolecules including VEGF, MMPs, and miRNAs (45). For instance, in breast cancer, EV-mediated delivery of miR-105 promotes metastasis by targeting the tight junction protein ZO-1, thereby increasing vascular permeability (46). Similarly, in lung cancer, EV miR-197-3p promotes metastasis by targeting TIMP2/3 in HUVECs, resulting in angiogenesis induction (47). A recent study in CRC reported that EV circTUBGCP4 induces CRC liver and lung metastasis by activating the AKT signaling pathway and tip cell formation (48). Furthermore, another study identified CRC-derived EV miR-221-3p as a key facilitator of endothelial cell angiogenesis by targeting suppressor of cytokine signaling 3. Of relevance to the clinic, miR-221-3p expression positively correlated with lymph node metastasis (49). In the current study, we have identified a mechanism whereby EV miR-6084 functions as an angiogenesis inhibitor by targeting *ANGPTL4*, which is a member of the ANGPTL family (50).

Hypoxia, a prevalent characteristic of solid tumors, significantly influences the dynamic interaction between cancer cells and the TME, thereby contributing to tumor progression and metastases (51). Cancer cells adeptly adapt to and thrive in hypoxic environments, exploiting them to foster tumor growth, angiogenesis, and metastasis (52). Notably, hypoxia has been shown to trigger the release of EVs from breast cancer cells, a process dependent on HIF1 or -2A. Mechanistically, HIFs directly bind to RAB22A, which is an essential protein involved in microvesicle budding from the plasma membrane. Intriguingly, RAB22A has been implicated in facilitating breast cancer metastasis in orthotopic mouse models (53). Moreover, hypoxia significantly orchestrates alterations in EV cargo and downstream signaling pathways within the TME (19, 54). In esophageal squamous cell carcinoma, H-EVs have been reported to promote proliferation, migration, invasion, and tube formation of HUVECs. These H-EVs were also shown to enhance the tumor growth and lung metastasis (22). Similarly, in CRC, H-EV–mediated delivery of miR-135a-5p targets Kupffer cells, establishing a hepatic PMN by regulating immunosuppression and cell adhesion, which ultimately promote CRC liver metastasis (55). Furthermore, other studies have unveiled the role of CRC-derived H-EVs in promoting metastasis by their transfer into normoxic CRC cells (56–58). In our present study, we have delineated the intricate interplay between CRC cells and HUVECs, elucidating that CRC H-EVs induce angiogenesis and liver metastasis. Subsequently, we identified the pivotal role of the reduced miR-6084 expression within H-EVs. ANGPTL4 is also closely correlated with multiple aspects of the TME, including angiogenesis, immune evasion, and metabolic reprogramming. For instance, in bladder cancer, the ANGPTL4/SDC1 signaling axis mediates the interaction between tumor cells and plasma cells, leading to poor immune response (59). In CRC, adipose-derived mesenchymal stem cells secrete TGFB1 to activate SMAD3 signaling in CRC cells, thereby promoting *ANGPTL4* transcriptional activation. This cascade drives glycolytic reprogramming and confers anoikis resistance, ultimately facilitating peritoneal metastasis (60). In this study, we have unveiled *ANGPTL4* as the direct target of miR-6084 in HUVECs, which influences the downstream JAK2/STAT3 pathway, finally driving CRC angiogenesis.

Circulating EV miRNAs have emerged as promising diagnostic and prognostic biomarkers for cancer (61, 62). For instance, serum EV miR-135a-5p levels were significantly elevated in patients with CRC displaying liver metastases (55). In our current study, we established that plasma-derived EV miR-6084 expression levels were significantly diminished in CRC patients with liver metastases when compared with those without metastatic disease. The findings from FISH analysis on tissue microarrays further validated a marked reduction in miR-6084 levels within cancer tissues of CRC patients with liver metastases. These results suggest that plasma-derived EV miR-6084 could serve as a valuable diagnostic indicator, particularly when conventional imaging methods encounter challenges in diagnosing liver metastases.

While our study has aimed to provide comprehensive insights, it is important to acknowledge its inherent limitations. First, the relatively small patient cohort in the tissue microarray analysis may have limited our ability to establish statistically significant correlations between lower miR-6084 expression levels and both overall survival and disease-free survival. Second, our miRNA microarray investigation was confined to the SW620 cell line, though we verified miR-6084 expression in SW480 and its derived EVs. Third, the main finding is reliant on in vitro and murine models, and whether these fully replicate human CRC metastasis deserves further discussion. Despite validating the angiogenesis-inhibiting role of miR-6084 in a CRC mouse model, our study’s scope remains confined. Further study is needed to address the mechanism in vivo. And the future investigations should explore whether miR-6084 expression correlates with additional phenotypes pertinent to CRC initiation and progression. Expanding these inquiries will offer a more comprehensive understanding of miR-6084’s involvement in CRC.

In conclusion, our study demonstrated that CRC H-EVs can induce angiogenesis and liver metastasis through the HIF1A/SP1/miR-6084/ANGPTL4 axis. Our findings also suggested that miR-6084 from plasma EVs of CRC may be used as a biomarker for prediction of liver metastasis. Also, it might serve as a potential molecule to treat CRC liver metastasis.

## Methods

### Sex as a biological variable.

This study involved male and female individual clinical specimens. Our limited human study did not have the power to test for sex effects. Male mice were used for all animal investigations. Sex was not considered as a biological variable.

### Cell culture.

Colon cancer cell lines SW620, HCT116, and SW480 cells were purchased from the cell bank of the Chinese Academy of Science (Shanghai, China), and HUVECs were purchased from Procell (Wuhan, China). SW620 cells were cultured under normoxic (20% oxygen concentration) or hypoxic conditions (1% oxygen concentration) in DMEM (Cytiva) plus 10% fetal bovine serum (FBS; Excell Bio) and 1% penicillin/ streptomycin (Beyotime) for 2 days. The EVs in FBS were removed by ultracentrifugation (120,000*g* for 16 hours). HUVECs were cultured in endothelial cell medium (ECM) containing 5% FBS and 1% endothelial cell growth factor (all purchased from Procell). All cells were cultivated in a humidified incubator at 37°C with 5% CO_2_. All cell lines were authenticated by short tandem repeat identification.

### EV isolation.

Ultrafiltration plus SEC were used to collect EVs (63). Briefly, cell supernatant fractions were collected and pelleted by centrifugation at 2,000*g* for 30 minutes. Then, the supernatant was collected and filtered with a 0.22 μm filter. Supernatants were then concentrated using a 100 kD ultrafiltration tube (MilliporeSigma) by centrifugation at 2,500*g* for 25 minutes. After that, concentrated supernatants were purified using Exosupur columns (Echo biotech) according to the manufacturers’ instructions. Plasma was first filtered with a 0.22 μm filter and purified using Exosupur columns. The isolated EVs were resuspended in PBS. The quantities of EVs for use in vivo and in vitro experiments were measured using a BCA Protein Assay Kit (Beyotime). The isolated EVs were divided in aliquots (only thawed once) and preserved at –80°C until use. All isolated EVs were used within 1 month.

### EV characterization.

NTA was performed using ZetaView PMX 110 (Particle Metrix) on 1 μL EV samples diluted 1,000-fold with PBS for analysis. The TEM was performed to investigate the morphological characteristics of EVs. Briefly, 50 μL EV samples were added to the sealing membrane. The copper mesh was covered on the sample. Next, 2% phosphotungstic acid solution was added to negatively stain samples for 3 minutes at room temperature. After drying at room temperature for 3 minutes, the dried samples were observed and photographed by TEM (JEM-1400FLASH, JEOL). Western blotting was also performed. Two positive marker antibodies (CD9, TSG101) and 3 negative marker antibodies (GRP94, Calnexin, APOA1) were used. We have supplied the information of all antibodies in Supplemental Table 3. Methods for Western blot are available in the following Western blot section.

### EV labeling and treatment.

For EV-tracking experiments, purified EVs were fluorescently labeled using DID membrane dye (MilliporeSigma). The method was referred to the manufacturer’s instruction. First, we added 5 μL of DID membrane dye to 1 mL EV suspension and mixed well. Next, we incubated at room temperature for 20 minutes. After that, we filtered once with a 0.22 μm MilliporeSigma filter membrane and stored at –20°C until use. Labeled EVs were injected into each mouse via tail vein. Twenty-four hours after injection, in vivo imaging and immunofluorescence were used to evaluate EV distribution in vivo and confirm the cells uptaking EVs. For in vitro assays, labeled EVs were added to the supernatant of HUVECs, and the EVs taken up by HUVECs at 1 hour, 3 hours, and 6 hours were captured by confocal microscopy (Leica Microsystems). DID membrane dye diluted by PBS was used as controls. For EV treatment assays, HUVECs were planted into 6-well plates 1 day before treatment. When the cells grew to about 70% confluence, 10 μg of N-EVs or H-EVs or equal volume of PBS (control) was added into cells. At 48 hours after treatment, cells were collected for the following experiments.

### GW4869 treatment.

To inhibit EV release, SW620 cells under normoxic or hypoxic condition were treated with GW4869 (Sigma-Aldrich), which is an inhibitor of EV formation and release, at a concentration of 10 μM for 48 hours. Then, supernatants were collected for EV isolation and subsequent experiments.

### Plasmid vector construction and cell transfection.

SP1 knockdown was performed using the shRNA system. Next, the recombinant pLKO.1-shSP1 vector was cotransfected with packing plasmids psPAX2 and PMD2.G into HEK293T cells. HEK293T was purchased from the cell bank of the Chinese Academy of Science (Shanghai, China). The viral supernatant was harvested to infect SW620 cells. Infected SW620 cells were selected with 2.5 μg/mL puromycin for 2 days. For ANGPTL4 overexpression, human ANGPTL4 genes were amplified by PCR and subcloned into vector CMV-EGFP-puro. For miR-6084 overexpression, pre–miR-6084 was composed and subcloned into vector CMV-EGFP-puro. Next, the recombinant plasmid CMV-EGFP-puro-ANGPTL4 was cotransfected with packing plasmids psPAX2 and PMD2.G into HEK293T cells. The viral supernatant was harvested to infect HUVECs. The shRNA oligos and primers for the overexpression vector construction were purchased from Tsingke Biological Technology Co., Ltd.

MiR-6084 mimics and inhibitors were obtained from RiboBio Company. HUVECs were seeded into 6-well plates 1 day before transfection. When they reached 70% confluence, HUVECs were transfected with miR-6084 mimics and inhibitors using Calcium Phosphate Cell Transfection Kit (Beyotime). ShRNA oligos and primers for the overexpression vector construction are listed in Supplemental Table 2.

### CCK8 assays.

Cell proliferation was assessed by CCK8. A total of 100 μL of cell suspension at a density of 3 × 10^4^/mL was incubated in a standard culture condition (37°C, 5% CO_2_ and 95% humidity) for 0 hour, 24 hours, and 48 hours. A total of 10 μL of CCK8 reagent (Beyotime) was added into each well, and then the cells were put back into the incubator and incubated for 4 hours. Absorbance at 450 nm was measured and read on a microplate reader (BioTek Instruments). Each assay was repeated at least 3 times. One representative of 3 independent experiments was shown.

### Wound healing assays.

Cells were counted and plated in a 6-well plate overnight. After making sure the cells covered the entire 6-well plate, the complete medium was replaced by serum-free medium to starve the cells for 24 hours. Subsequently, a 200 μL pipette tip was used to scratch 3 straight lines in the cell monolayer. Cell migration was observed under a Nikon Eclipse Ti inverted microscope after another 24 hours, and cell migration distance was measured. Each assay was repeated at least 3 times. One representative of 3 independent experiments was shown.

### Transwell migration assays.

A total of 100 μL 2% FBS ECM containing 5 ×10^4^ HUVECs was seeded into the upper chamber of Transwell chambers (24 wells) (Merck). A total of 600 μL of the 10% FBS ECM was seeded into the lower chamber. The bottom surface of the membranes was fixed with paraformaldehyde after 24 hours, stained with crystal violet, and photographed. The number of migrating cells was counted in 3 random microscopic fields under the Nikon inverted microscope. Each assay was repeated at least 3 times. One representative of 3 independent experiments was shown.

### Tube formation assays.

A total of 50 μL of Matrigel matrix (Corning) was plated in a 96-well plate and incubated at 37°C for 30 minutes to allow the Matrigel to polymerize. The treated HUVECs were counted, and 2.5 × 10^4^ HUVECs were seeded on the Matrigel-coated well. Then, the plate was incubated at 37°C. Tube formation was observed at 12 hours with a microscope after staining with Calcein-AM (Beyotime). The tube formation ability was determined by measuring the total length of tubes. Each assay was repeated at least 3 times. One representative of 3 independent experiments was shown.

### RT-qPCR assays.

Total RNA from cell was isolated using a Cell Total RNA Isolation Kit (Foregene). Reverse transcription was conducted using the PrimeScript RT reagent Kit (Takara) according to the manufacturer’s protocol. RT-qPCR was performed using a CFX96 Real-Time PCR System (Bio-Rad) with SYBR Green Supermix (Novoprotein). The relative expression levels of target genes were normalized to β-actin. Total RNA from EVs was isolated using an miRNeasy Mini Kit (QIAGEN). Reverse transcription was conducted using the Mir-X miRNA First-Strand Synthesis Kit (Takara). The relative expression levels of target genes were normalized to U6. The primers for RT-qPCR used in this study are in Supplemental Table 4.

### Western blot.

The isolated EVs or cell precipitate was suspended in RIPA lysis buffer and incubated on ice for 10 minutes. Then the total protein concentration was measured by a BCA Protein Assay Kit. An appropriate amount of 5× protein loading buffer was added to the protein sample and then heated to 99°C for 10 minutes. A total of 20 μg protein loaded in each lane was separated on SDS-PAGE and then transferred onto PVDF membranes (Bio-Rad). Then, after blocking with 5% skim milk powder for 2 hours at room temperature, the membranes were cut and incubated in primary antibody overnight at 4°C. EV proteins (CD9, TSG101, GRP94, Calnexin) were detected with the antibodies. All primary antibodies were used at a diluted concentration based on the manufacturer’s protocol (Supplemental Table 3). After being washed 5 times in TBS with Tween, membranes were incubated with a secondary antibody (HRP-conjugated Goat Anti-Rabbit IgG, Proteintech, SA00001-2; HRP-conjugated Goat Anti-Mouse IgG, Proteintech, SA00001-1) for 2 hours at room temperature. After another 5 times of washing in PBS, the immunostaining intensity of each protein band was quantified using a Bio-Rad ChemiDoc XRS system.

### MiRNA microarray.

Affymetrix miRNA 4.0 microRNA microarray was used. SW620 cell supernatant was collected, and EVs were isolated by ultrafiltration plus SEC. Then further RNA isolation, adding Poly A tail and biotin labeling, hybridization, washing, staining, and scanning were performed by Echo biotech (Beijing, China) according to the Affymetrix protocol.

### Immunofluorescence.

SW620 cells or HUVECs were plated on glass coverslips a day in advance. First, the treated cells were fixed with 4% paraformaldehyde for 30 minutes. Next, the cells were permeabilized with 0.5% Triton X-100 and blocked with 3% bovine serum albumin for 30 minutes. Then, primary antibodies were incubated on a shaker at 4°C overnight. After that, secondary antibodies (CoraLite488-conjugated Goat Anti-Rabbit IgG, Proteintech, SA00013-2; or CoraLite594-conjugated Goat Anti-Rabbit IgG, Proteintech, SA00013-4) were incubated at 37°C for 1 hour. The nuclei were stained with DAPI for 5 minutes and the anti-fluorescence quencher was used to seal the sections. Finally, images were captured using a confocal laser scanning microscope (Leica Microsystems Inc.).

### Dual luciferase reporter assays.

HUVECs (5 ×10^5^) were transfected with 1 μg of the pmiR-RB-REPORT-ANGPTL4 3′-UTR-wt or pmiR-RB-REPORT-ANGPTL4 3′-UTR-mut plasmids (RiboBio) using Calcium Phosphate Cell Transfection Kit (Beyotime). These plasmids contain both firefly luciferase and renilla luciferase genes. In addition, 1 μg mimic-NC or mimic-6084 was cotransfected into HUVECs. Dual Luciferase Reporter Gene Assay Kit (Beyotime) was used to detect the luciferase activity after 48 hours.

### ChIP.

ChIP assays were performed according to a protocol in a previous study (64). Briefly, SW620 cells were cross-linked with 37% formaldehyde, and genomic DNA was sheared to 200–1,000 bp by a sonicator. Ten percent of the lysate was kept as input. Then, the remaining lysate was incubated with the SP1 antibody in a rotator overnight. Next, Protein A/G Magnetic Beads (MedChemExpress) were added and incubated with above complex for 3 hours. Proteinase K was added to de-cross-link the precipitated DNA at 65°C. After that, the complex was purified with a DNA Fragment Purification Kit (Takara). Last, the products were analyzed by RT-qPCR. Primers specific to ChIP are listed in Supplemental Table 5.

### IP.

SW620 cells were washed twice with PBS and lysed with lysis buffer (20 mmol/L Tris-HCl pH 7.4, 150 mmol/L NaCl, 1% NP-40, 1 mmol/L EDTA, and 5% glycerol) for 30 minutes. After removing cell debris by centrifugation (12,000*g*, 20 minutes), the supernatant was incubated with specific antibodies in a rotator overnight. Then, Protein A/G Magnetic Beads (MedChemExpress) were added to bind the above complex for 2 hours at in a rotator. After 5 times of washing in lysis buffer, protein A/G beads were diluted with 1× protein loading buffer and heated to 99°C for 10 minutes. Last, samples were detected by Western blot.

### Clinical samples.

Blood samples and the sample of human primary CRC tissues were obtained from the West China Hospital of Sichuan University (Chengdu, China). For blood samples, plasma was obtained through centrifugation (2,000*g*, 10 minutes), and then ultrafiltration plus SEC were used to collect EVs. The baseline characteristics of included patients are listed in Supplemental Table 6. Tissue samples were collected from surgically resected colorectal tissue and then placed into 4% paraformaldehyde immediately. Further RNA-FISH and IHC assays were performed to detect miR-6084 and SP1 expression.

### Tissue microarray.

The tissue microarray was purchased from Zhuoli Biotechnology Co. Ltd. It contains 80 pairs of primary CRC tissues, including 20 pairs of tissues from CRC with liver metastasis patients. Further RNA-FISH and IHC assays were performed to detect miR-6084 and SP1 expression. Briefly, for FISH, tissue samples were fixed, dehydrated, paraffin-embedded, and sectioned. Sections were deparaffinized, rehydrated, and subjected to antigen retrieval and digestion. Prehybridization was performed to block nonspecific sites, followed by hybridization with miR-6084–specific probes. Nuclei were counterstained with DAPI. For IHC, paraffin-embedded tissue sections were deparaffinized and rehydrated. Antigen retrieval was performed to unmask epitopes, followed by blocking of endogenous peroxidase activity. Sections were incubated with serum to prevent nonspecific binding, then treated with primary antibodies specific to SP1 proteins. Secondary antibodies conjugated with HRP were applied (HRP-conjugated Goat Anti-Rabbit IgG, Proteintech, SA00001-2), and DAB substrate was used for chromogenic detection. Nuclei were counterstained by hematoxylin. Image preprocessing was conducted by CaseViewer 2.3 (3DHISTECH).

### Animal studies.

For EV uptake assays, male BALB/c nude mice (6–8 weeks) were purchased from HUAFUKANG and housed in a pathogen-free animal facility. DID^+^ EVs were injected through the tail vein for 30 minutes and 24 hours. Then the distribution of EVs was observed by in vivo imaging using the AniView 100 Living Imaging software (BLT). Then, mice were sacrificed and their liver, lungs, heart, spleen, kidneys, gastrointestinal tract, and femurs were imaged in vitro.

For liver premetastasis and metastasis models, male BALB/c nude mice (6–8 weeks) were purchased from HUAFUKANG and housed in a pathogen-free animal facility. To evaluate the role of EVs on distant liver metastasis, mice were first pre-educated with SW620-derived EVs. We injected 10 μg EVs into each mouse every day via the tail vein. After 2 weeks of education, FITC-Dextran was injected into some mice via the tail veins, these mice were sacrificed 0.5 hour later, and livers were collected for immunofluorescence to reflect the vascular permeability and neovascular density in the liver PMN. At the same time, some mice were injected intrasplenically with 4 × 10^6^ luciferase-labeled SW620 cells to establish liver metastatic models. To avoid the interference of fluorescence from tumor cells in the spleen, two-thirds of the spleen was resected 3 minutes later after the tumor cell injection. Then, liver metastasis was detected by ex vivo, luciferase-based, noninvasive bioluminescence imaging. Mice were sacrificed 2 weeks later and livers were collected. Liver metastasis was quantified by the number of nodules on the liver surface.

For Matrigel plug-in assays, male BALB/c nude mice (6–8 weeks) were purchased from HUAFUKANG and housed in a pathogen-free animal facility. Matrigel (400 μL, BD Biosciences) mixed with 10 μg (100 μg/mL) EVs was injected subcutaneously into the dorsal region of BALB/c nude mice. Mice were sacrificed 10 days later, and the Matrigel plugs were dissected. The plugs were stained with H&E and Masson’s. CaseViewer (version 2.3.0) was used for image acquisition. Three mice were used for each group. One representative of 3 independent experiments was shown.

### Statistics.

The statistical analyses were performed by SPSS software version 22.0 (IBM) and GraphPad Prism 8.0 software. Continuous variables were expressed by the mean with SD. The unpaired 2-tailed Student’s *t* test was used to test statistical significance between the 2 groups, and 1-way ANOVA was used for statistical analysis of more than 2 groups. A *P* < 0.05 was considered statistically significant.

### Study approval.

All patients gave informed written consent with the approval of the Biological and Medical Ethics Committee of West China Hospital of Sichuan University (Chengdu, China, IRB number 2023-2112). All animal experimental procedures were in accordance with protocols approved by the Experimental Animal Ethics Committee of West China Hospital, Sichuan University (Chengdu, China, Ethics record number 20220107010).

### Data availability.

The manuscript and the supplement present all the data needed to evaluate the study’s conclusions. All original data sets and analyses, including individual comparisons, are also reported in the Supporting Data Values file. All data sets are available on the NCBI GEO with the entry number GSE297674.

## Author contributions

Yang Zhang, XY, and Yaguang Zhang designed and conducted experiments, and Yang Zhang wrote the manuscript. SZ and QH provided technical guidance on ChIP and Co-IP assays. SL performed data analysis. LQ, MW, and XD provided technical guidance on animal experiments. WM and HC helped design experiments. Review and editing were performed by JH and ZW. All authors have read and agreed to the published version of the manuscript.

## Supplementary Material

Supplemental data

Unedited blot and gel images

Supporting data values

## Version 1 06/10/2025

In-Press Preview

## Version 2 07/22/2025

Electronic publication
