# Supplementary material for: MicroRNA-6084 orchestrates angiogenesis and liver metastasis in colorectal cancer via extracellular vesicles
Source: JCI Insight. 2025 Jun 10;10(14):e189503. doi: 10.1172/jci.insight.189503 (PMC12288965; doi:10.1172/jci.insight.189503)
Supplement: Supplemental data [file jciinsight-10-189503-s041.pdf]

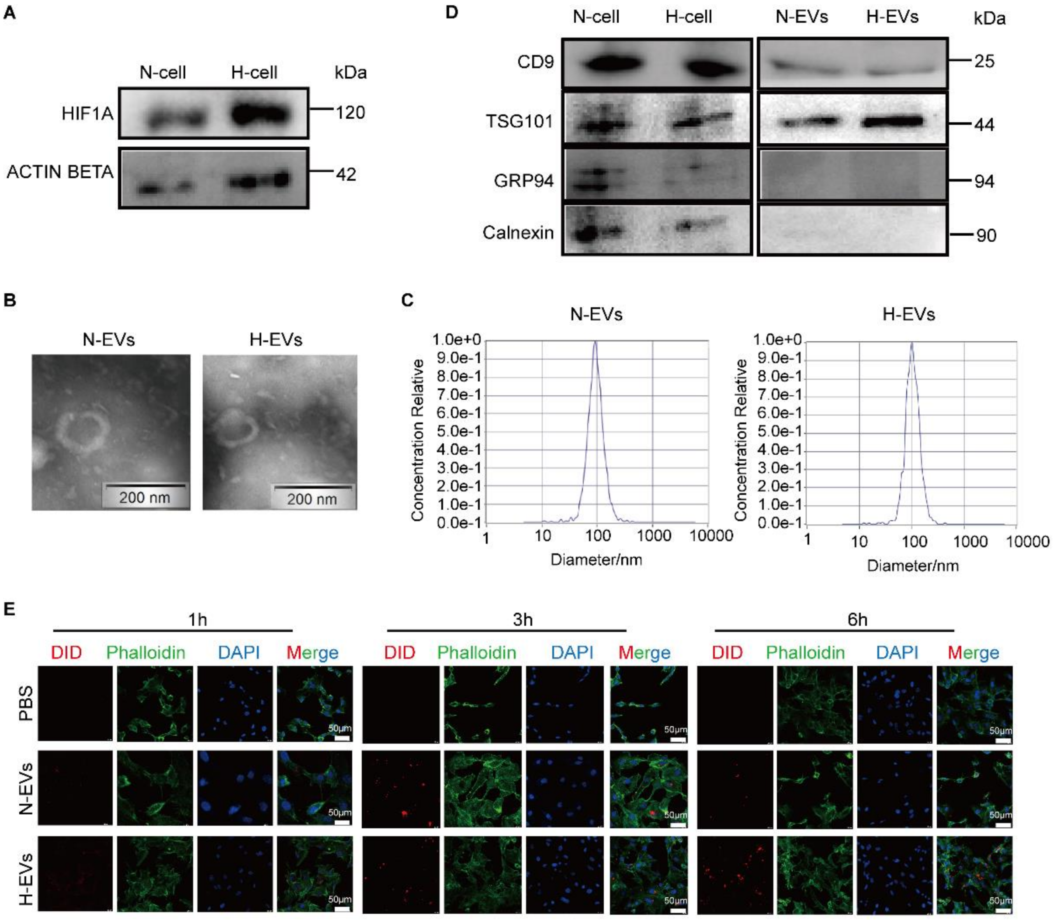

Supplementary Figure 1. The characterization of SW620-derived EVs. A. The expression of HIF1A in SW620 cells under normoxic (N-cell) and hypoxic (H-cell) conditions. **B.** Transmission electron microscope (TEM) images of EVs. **C.** The representative nanoparticle tracking analysis (NTA) of EVs. **D.** The EV positive (CD9 and TSG101) and negative (GRP94, Calnexin) markers were analyzed by western blot. **E.** Uptake of EVs derived from normoxic SW620 or hypoxic SW620 by HUVECs at 1 h, 3 h, 6 h. Fluorescence microscopy images showing the internalization of EVs by HUVECs. Blue: Nucleus stained with DAPI. Red: DID-labeled EVs. Green: CoraLite®488 Phalloidin. Scale bar, 20μm. N-EVs: EVs isolated from SW620 under normoxia. H-EVs: EVs isolated from SW620 under hypoxia.

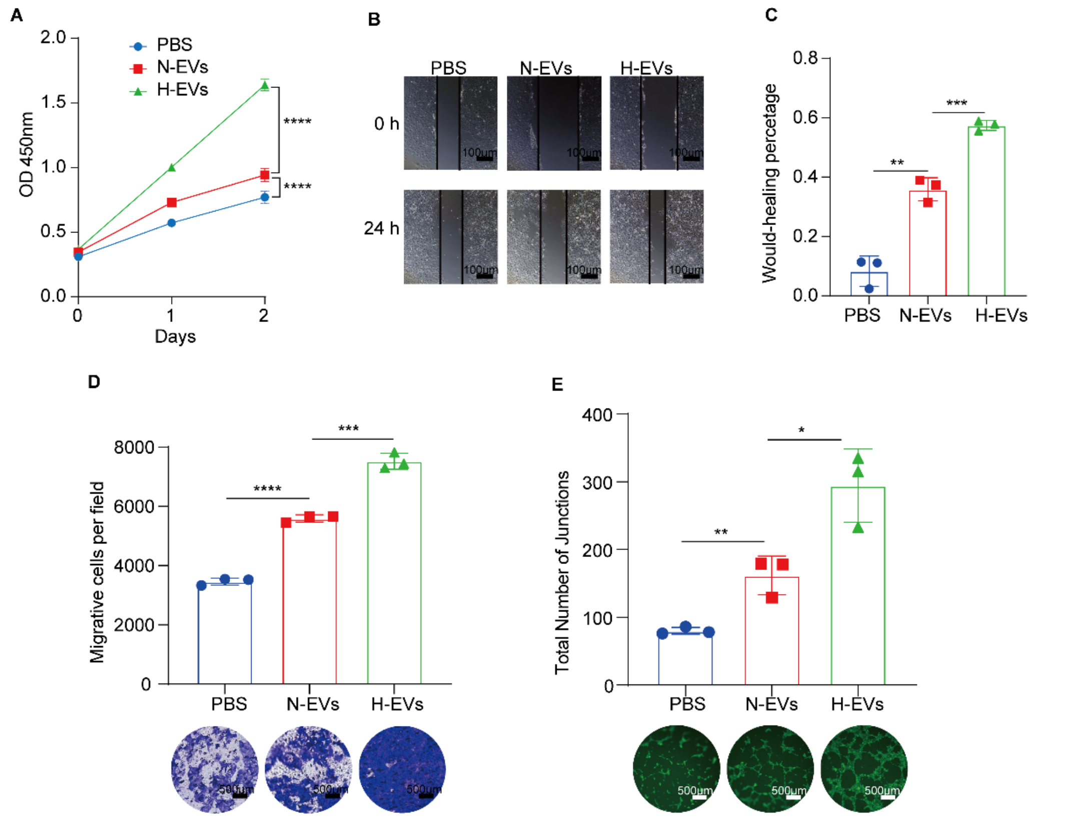

Supplementary Figure 2. The impact of PBS, HCT116 derived normoxic EVs (N-EVs), and hypoxic EVs (H-EVs) in the proliferation, migration, tube formation of HUVECs. A. The proliferation ability of HUVECs treated with PBS, N-EVs, and H-EVs were analyzed by CCK8 assay. The migration ability was assessed by would healing assay **(B)** and transwell assay (**D**). The relative migration distance (%) was calculated (**c**). The number of migrative cells was counted and graphed (**D**). Representative pictures of tube formation were taken after stained with Calcein-AM and quantified by measuring the total vessel length (**E**). Statistical significance was assessed with 1-way ANOVA with Tukey's multiple-comparison test (**A**, **C-E**), * $P < 0.05$, ** $P < 0.01$, *** $P < 0.001$, **** $P < 0.0001$.

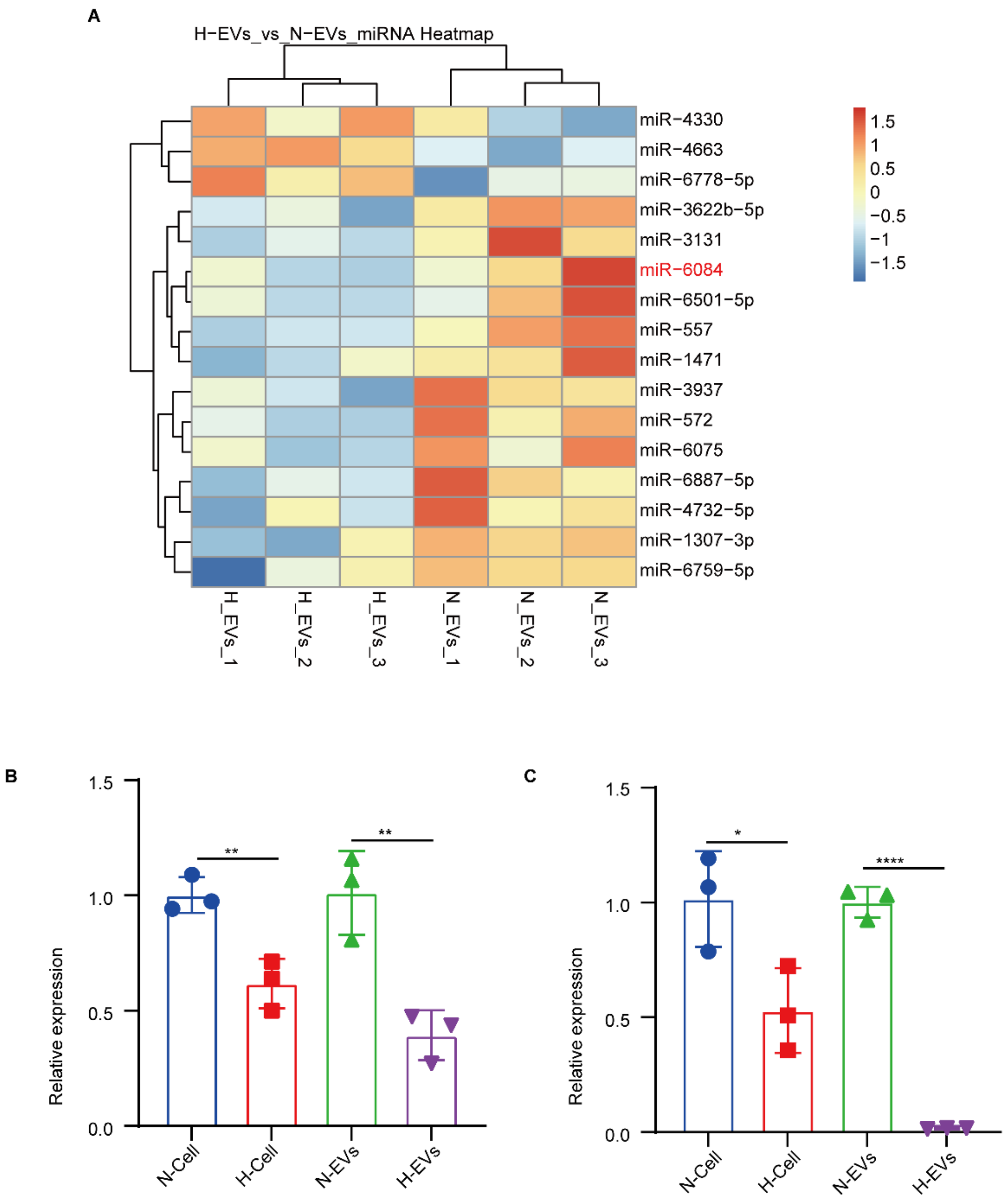

Supplementary Figure 3. Selection of differentially expressed miRNAs between normoxic and hypoxic EVs. A. Heat maps visually display differentially expressed genes (|FC|>1.5). **B.** The expression of miR-6084 was validated using RT-qPCR in SW620 and derived EVs. **C**. The expression of miR-6084 was validated using RT-qPCR in SW480 and derived EVs. U6 was used as internal control. Each experiment was conducted at least 3 times. Data was presented as mean ± standard deviation (SD). Statistical significance was assessed with 1-way ANOVA with Tukey's multiple-

comparison test (**B-C**), * $P < 0.05$, ** $P < 0.01$, **** $P < 0.0001$.

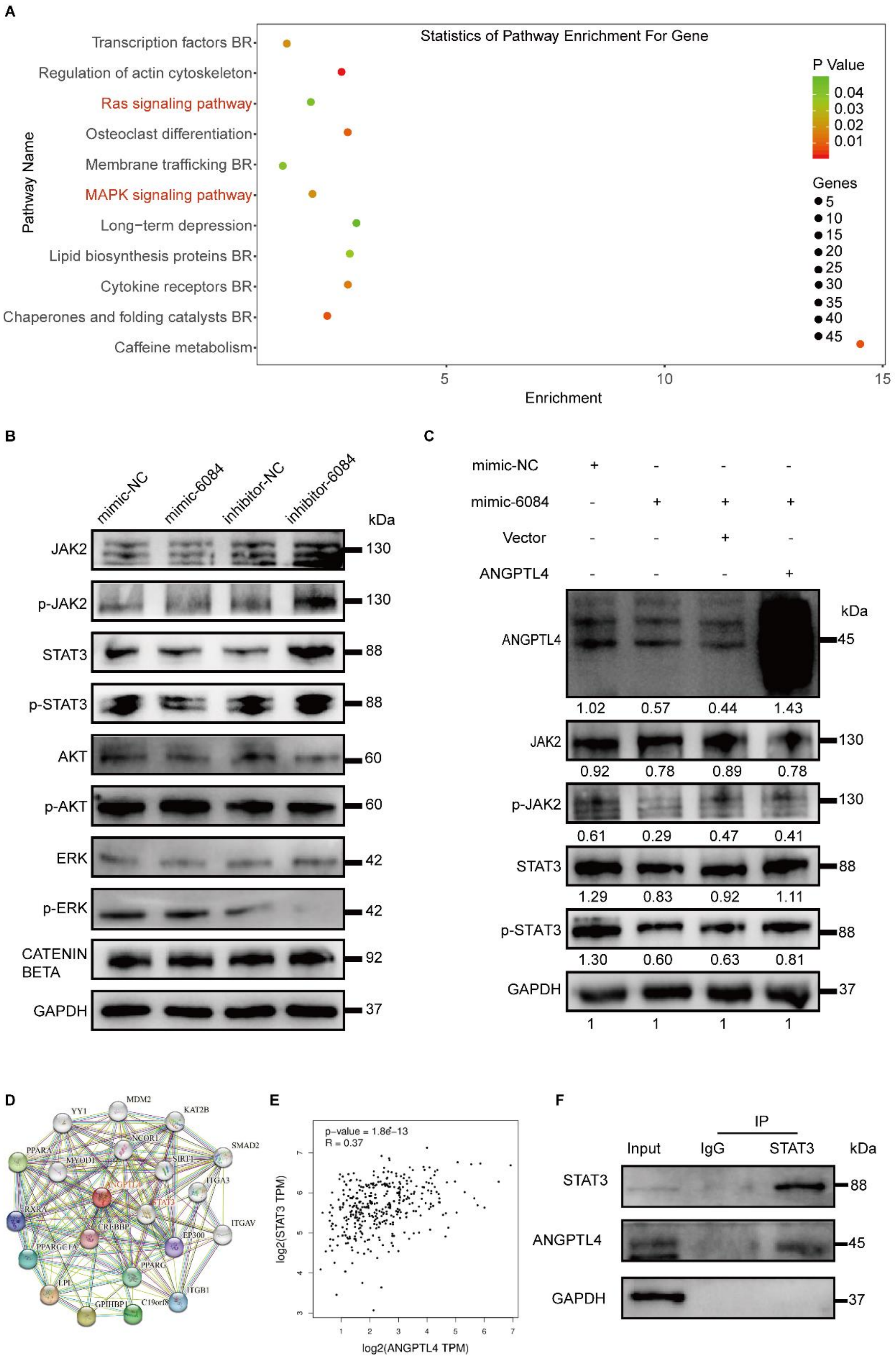

Supplementary Figure 4. MiR-6084 inhibits angiogenesis through ANGPTL4 mediating JAK2/STAT3 pathway. A. The KEGG pathway enrichment analysis of the

regulated genes of miR-6084. **B.** Key proteins of several common pathways (JAK2/STAT3, PI3K/AKT, RAS/RF/ERK/MAPK, Wnt/CATENIN BETA) were detected by Western blot. **C.** ANGPTL4 can rescue the inhibited JAK2/STAT3 pathway. **D.** The protein-protein interaction (PPI) network was conducted by STRING (https://string-db.org). **E.** The expression correlation of ANGPTL4 and STAT3 was performed by GEPIA2. **F.** Immunoprecipitation experiments were preformed to confirmed the interaction between ANGPTL4 and STAT3.

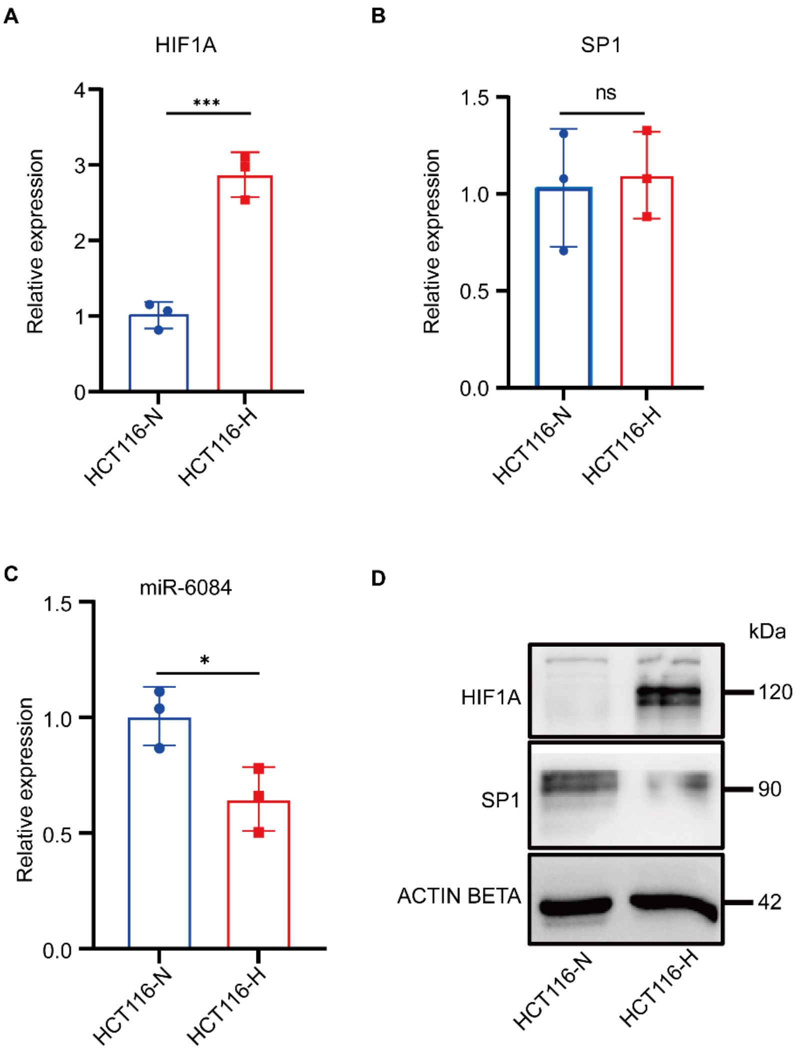

Supplementary Figure 5. QPCR and western blot were conducted to reveal the mutual regulatory relationship between HIF1A and SP1 in HCT116 cell line. A. Expression level of *HIF1A* mRNA in HCT116 under normoxic or hypoxic condition detected by qPCR. **B.** Expression level of *SP1* mRNA in HCT116 under normoxic or hypoxic condition detected by qPCR. **C.** Expression level of miR-6084 in HCT116 under normoxic or hypoxic condition detected by qPCR. **D.** Expression level of HIF1A and SP1 protein in HCT116 under normoxic or hypoxic condition detected by western blot. Statistical significance was assessed with 2-tailed unpaired Student's t test (**A-C**), ns represents no significant difference, * $P < 0.05$, *** $P < 0.001$.

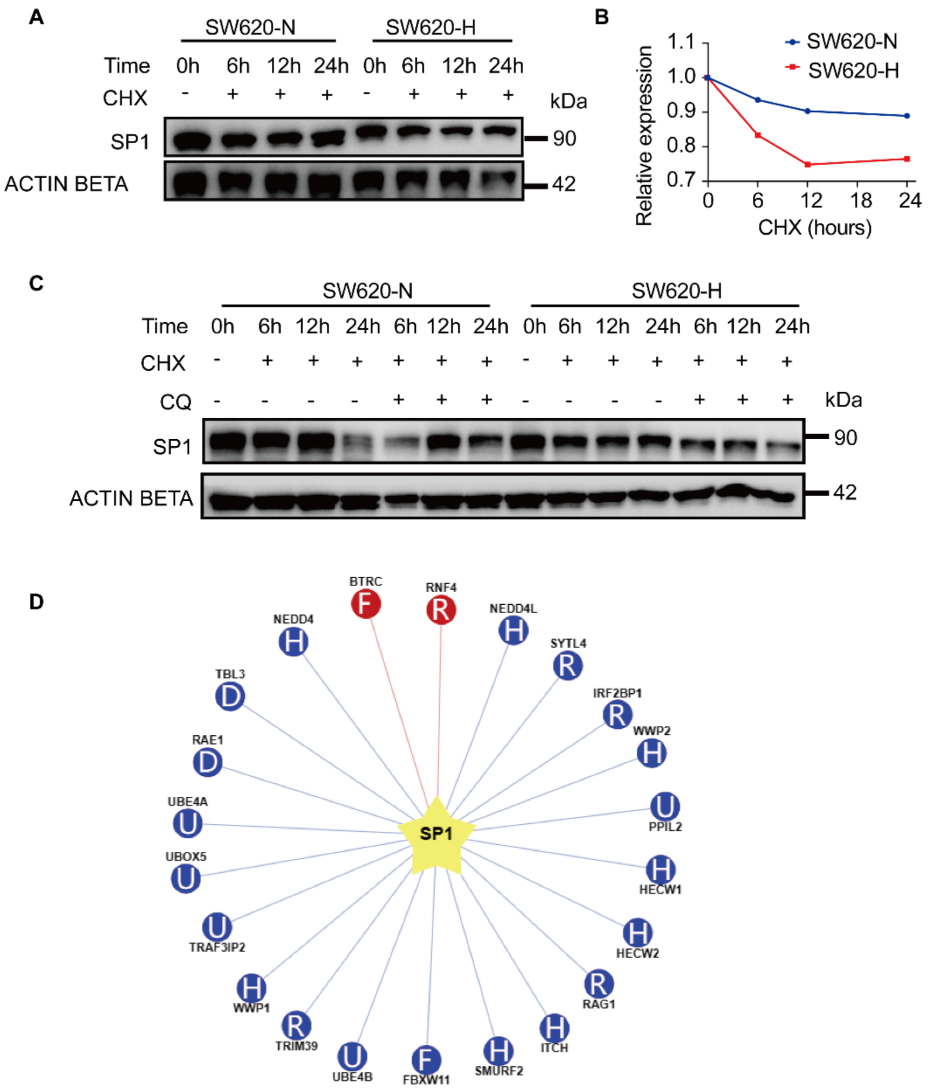

Supplementary Figure 6. HIF1A modulates miR-6084 expression by regulating SP1 protein ubiquitination and proteasomal degradation. A. SW620 cells were treated by CHX (a protein synthesis inhibitor), then SP1 protein stability was measured by western blot. **B.** Half-life curves were drawn according to panel A. **C.** SW620 cells were treated by CHX (a protein synthesis inhibitor) and CQ (chloroquine, an autophagy inhibitor), then SP1 protein stability was measured by western blot. **D.** Predicted E3 ubiquitin ligase of SP1 in UbiBrowser, an integrated bioinformatics platform (http://ubibrowser.ncpsb.org).

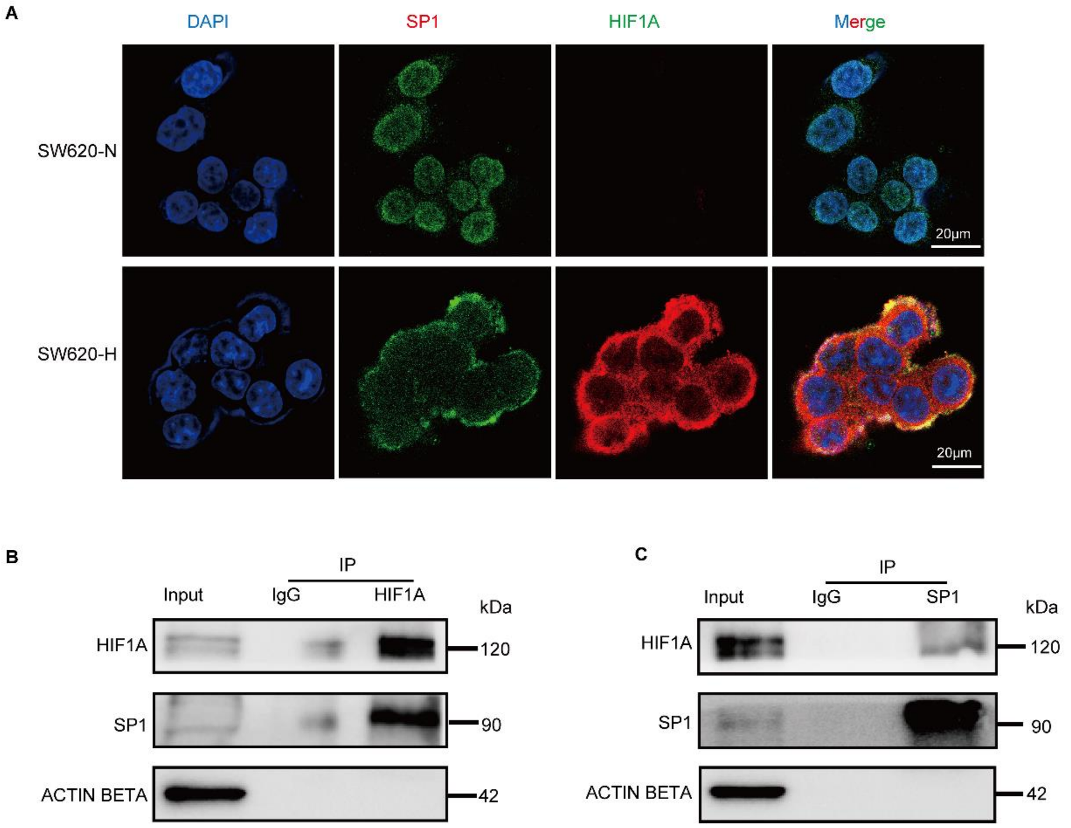

Supplementary Figure 7. HIF1A modulates miR-6084 expression by sequestering SP1 from the miR-6084 promoter. A. Representive images of immunofluorescence revealing the colocalization of SP1 and HIF1A. **B-C**. Co-immunoprecipitation assays were conducted to confirm the binding between SP1 and HIF1A.

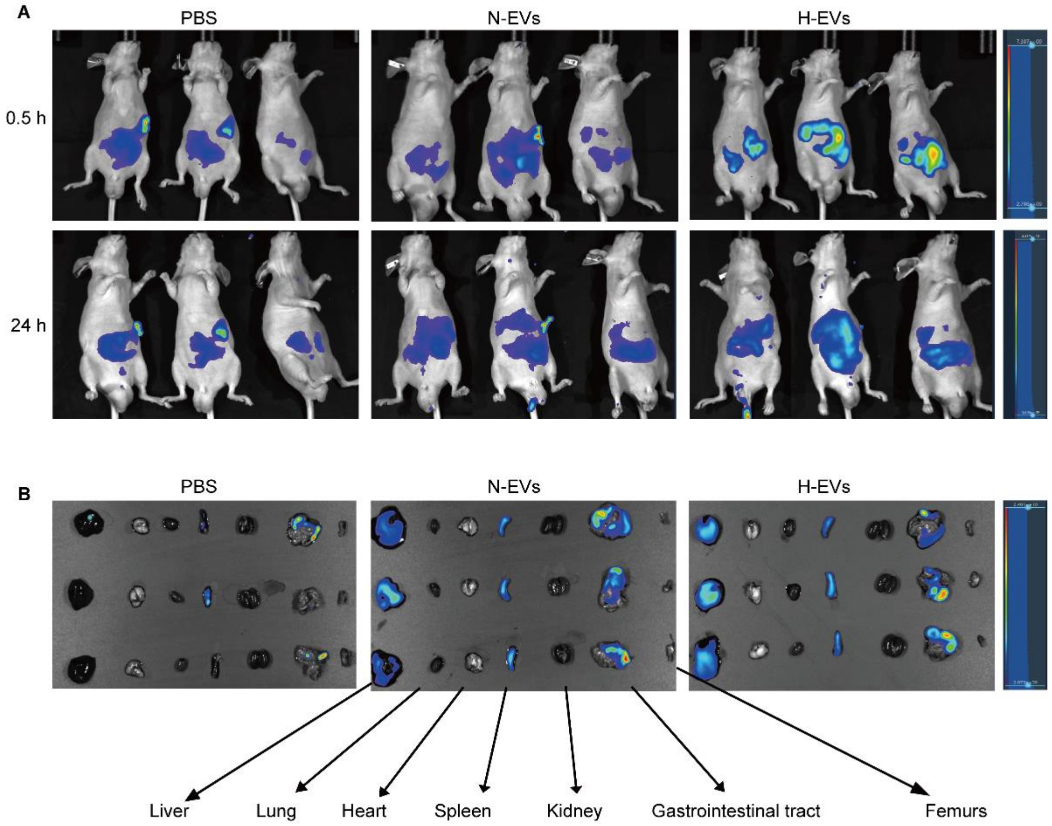

Supplementary Figure 8. Uptake of normoxic or hypoxic SW620 derived EVs by nude mice. A. DID+ EVs were injected through the tail vein for 30min and 24 hours. Then the distribution of EVs was observed by in vivo imaging. **B.** Mice were sacrificed and their liver, lungs, heart, spleen, kidneys, gastrointestinal tract and femurs (from left to right) were imaged in vitro.

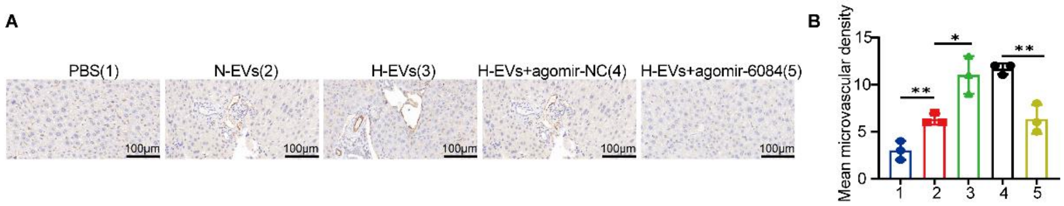

Supplementary Figure 9. CRC-derived H-EVs promote angiogenesis and liver metastasis through EV miR-6084 in vivo. **A**. Immunohistochemistry staining (IHC) for CD31 of liver tissues in each group. **B**. Microvascular density was counted. Each experiment was conducted at least 3 times. Data was presented as mean ± standard deviation (SD). Statistical significance was assessed with 1-way ANOVA with Tukey's multiple-comparison test (**B**), * $P < 0.05$, ** $P < 0.01$.

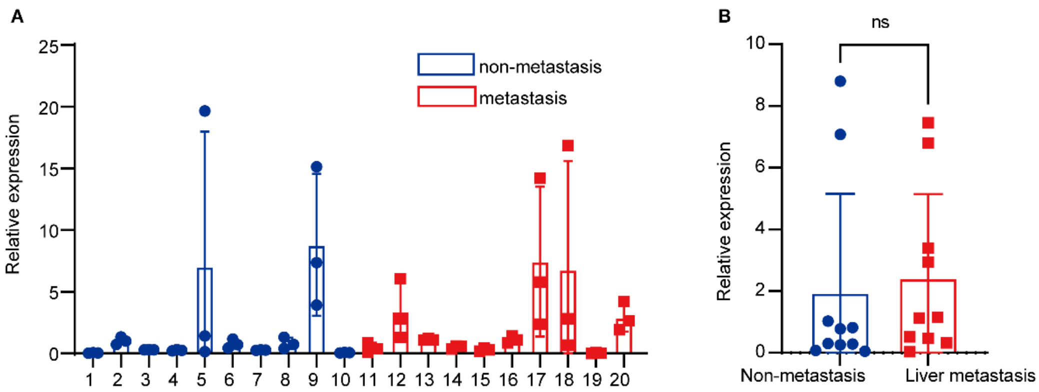

Supplementary Figure 10. Plasma miR-6084 is comparable between CRC patients with or without liver metastasis. **A**. Levels of miR-6084 in the plasma of CRC patients with or without liver metastasis detected by qPCR. **B**. Unpaired t-test was performed between non-metastasis group and liver metastasis group. Statistical significance was assessed with 2-tailed unpaired Student's *t* test (**B**), ns represents no significant difference.

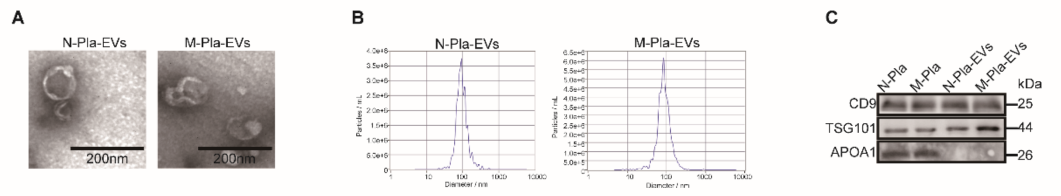

Supplementary Figure 11. The characterization of plasma-derived EVs. A. Transmission electron microscope (TEM) images of EVs. B. The representative nanoparticle tracking analysis (NTA) of EVs. C. The EV positive (CD9 and TSG101) and negative (APOA1) markers were analyzed by western blot. N, non-metastasis; M, liver metastasis.

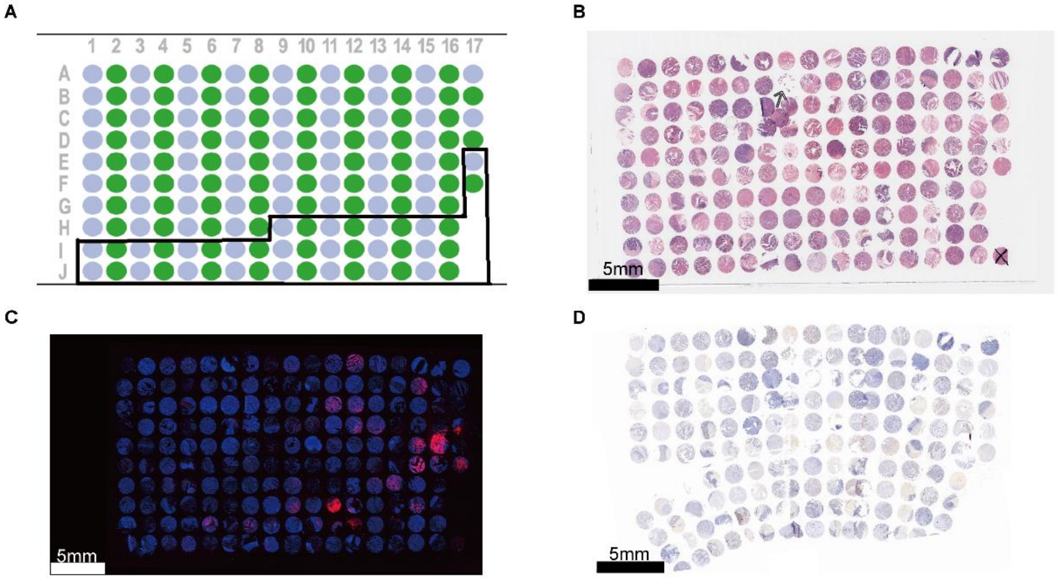

E

	miR-6084 low	miR-6084 high	*p* value
**SP1**			0.008
**Low**	15 (36.6)	28 (66.7)	
**high**	26 (63.4)	14 (33.3)	

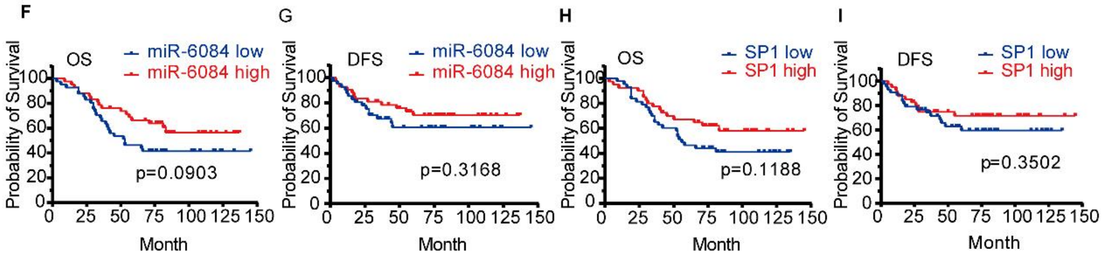

Supplementary Figure 12. The complete information of CRC tissue chips. A. The schematic diagram of each point in the tissue chips (the blue spots refer to cancerous tissues, the green spots refer to adjacent tissues and spots in black bar box refer to patients with liver metastasis). **B.** The complete image of H.E staining in the tissue chips. **C.** The whole image of miR-6084 ISH in CRC tissue chips. **D.** The complete image of SP1 IHC in the CRC tissue chips. **E.** Chi-square test was performed to analyze correlation between miR-6084 and SP1. **F.** Overall survival curves of CRC patients with miR-6084 low/high expression. **G.** Disease-free survival curves of CRC patients with miR-6084 low/high expression. **H.** Overall survival curves of CRC patients with SP1 low/high expression. **I.** Disease-free survival curves of CRC patients with SP1 low/high

expression. Estimation of the relationship between miR-6084/SP1 expression and OS/DFS times in CRC patients was performed by Kaplan-Meier analysis, *P* value was calculated with log-rank test.

Supplementary Table 1. The detailed information of these 16 differently expressed miRNAs

Hypoxsia	Normaxia	FC	Regulation	P.Value	Accession	Transcript.ID.Array.Design.	Chromosome	Start	Stop	Strand	Length	Sequence
2.13	3.07	**-1.919**	down	0.0086492	MIMAT0005951	**hsa-miR-1307-3p**	chr10	105154058	105154079	-	22	ACUCGGCGUGGCGUCGGUCGUG
0.27	1.16	**-1.853**	down	0.0064324	MIMAT0018352	**hsa-miR-3937**	chrX	39520530	39520552	+	23	ACAGGCGGCUGUAGCAAUGGGGG
0.21	1.06	**-1.803**	down	0.0333304	MIMAT0023709	**hsa-miR-6084**	chr1	20960249	20960268	+	20	UUCCGCCAGUCGGUGGCCGG
0.51	1.33	**-1.765**	down	0.0353055	MIMAT0019855	**hsa-miR-4732-5p**	chr17	27188718	27188740	-	23	UGUAGAGCAGGGAGCAGGAAGCU
1.62	2.44	**-1.765**	down	0.0389673	MIMAT0027418	**hsa-miR-6759-5p**	chr12	58142439	58142460	-	22	UUGUGGGUGGGCAGAAGUCUGU
0.22	1.03	**-1.753**	down	0.0049175	MIMAT0003221	**hsa-miR-557**	chr1	168344822	168344844	+	23	GUUUGCACGGGUGGGCCUUGUCU
-0.01	0.79	**-1.741**	down	0.008153	MIMAT0027674	**hsa-miR-6887-5p**	chr19	35613609	35613631	+	23	UGGGGGGACAGAUGGAGAGGACA
2.51	3.27	**-1.693**	down	0.0163286	MIMAT0007349	**hsa-miR-1471**	chr2	232756987	232757008	-	22	GCCCGCGUGUGGAGCCAGGUGU
0.32	1.01	**-1.613**	down	0.0042973	MIMAT0018005	**hsa-miR-3622b-5p**	chr8	27559243	27559262	-	20	AGGCAUGGGAGGUCAGGUGA
0.45	1.13	**-1.602**	down	0.0102543	MIMAT0014996	**hsa-miR-3131**	chr2	219923447	219923469	-	23	UCGAGGACUGGUGGAAGGGCCUU
0.27	0.94	**-1.591**	down	0.0040383	MIMAT0003237	**hsa-miR-572**	chr4	11370511	11370530	+	20	GUCCGCUCGGCGGUGGCCCA
0.39	1.05	**-1.58**	down	0.0486953	MIMAT0025458	**hsa-miR-6501-5p**	chr21	34922970	34922991	+	22	AGUUGCCAGGGCUGCCUUUGGU
0.22	0.83	**-1.526**	down	0.0260276	MIMAT0023700	**hsa-miR-6075**	chr5	1510882	1510902	-	21	ACGGCCCAGGCGGCAUUGGUG
2.49	1.79	**1.625**	up	0.0115106	MIMAT0027456	**hsa-miR-6778-5p**	chr17	18244174	18244195	-	22	AGUGGGAGGACAGGAGGCAGGU
2.79	1.53	**2.395**	up	0.0465264	MIMAT0016924	**hsa-miR-4330**	chrX	150336770	150336788	+	19	CCUCAGAUCAGAGCCUUGC
2.04	0.51	**2.888**	up	0.0005327	MIMAT0019735	**hsa-miR-4663**	chr8	124228071	124228094	-	24	AGCUGAGCUCCAUGGACGUGCAGU

Supplementary Table 2. ShRNA oligos and primers for the overexpression vector construction.

Primer name	Sequence
shSP1-F	5′-CCGGGCTGGTGGTGATGGAATACATCTCGAGATGTATTCCATCACCACCAGCTTTTTG-3′
shSP1-R	5′-AATTCAAAAAGCTGGTGGTGATGGAATACATCTCGAGATGTATTCCATCACCACCAGC-3′
ANGPTL4-F	5′-ggatctatttccggtgaattcATGAGCGGTGCTCCGACG-3′
ANGPTL4-R	5′-caacttttgttccatgaattcCTAGGAGGCTGCCTCTGCTG-3′

Supplementary Table 3. List of primary antibodies used in this study.

Antibody	Company	Catalog	Experiment	Dilution
CD9	Abcam	ab236630	Western Blot	1:1000
TSG101	Abcam	ab125011	Western Blot	1:1000
GRP94	Abcam	ab238126	Western Blot	1:1000
Calnexin	Abcam	ab22595	Western Blot	1:1000
HIF1A	Proteintech	20960-1-AP	Western Blot	1:1000
HIF1A	Proteintech	20960-1-AP	Immunoprecipitation	1:300
HIF1A	CST	79233T	Immunofluorescence	1:200
ACTIN BETA	Proteintech	66009-1-Ig	Western Blot	1:1000
JAK2	Abcam	ab108596	Western Blot	1:1000
p-JAK2	Abcam	ab32101	Western Blot	1:1000
STAT3	Abcam	ab68153	Western Blot	1:1000
p-STAT3	Abcam	ab267373	Western Blot	1:1000
AKT	Abcam	ab8808	Western Blot	1:1000
p-AKT	Abcam	ab38449	Western Blot	1:1000
ERK	HUABIO	ET1601-29	Western Blot	1:1000
p-ERK	HUABIO	ET1610-13	Western Blot	1:1000
GAPDH	Proteintech	60004-1-Ig	Western Blot	1:1000
ANGPTL4	Proteintech	18374-1-AP	Western Blot	1:1000
SP1	Proteintech	21962-1-AP	Western Blot	1:1000
SP1	Proteintech	21962-1-AP	Immunoprecipitation	1:300
SP1	Proteintech	21962-1-AP	Immunofluorescence	1:50
SP1	Proteintech	21962-1-AP	IHC	1:300
SP1	CST	9389S	CHIP	1:100
p-SP1^{T453}	Affinity	AF3121	Western Blot	1:1000

p-SP1^{T739}	Affinity	AF3122	Western Blot	1:1000
CD31	Proteintech	28083-1-AP	IHC	1:8000
APO1	Proteintech	14427-1-AP	Western Blot	1:1000
BTRC	CST	4394T	Western Blot	1:1000
Ubiquitin	CST	20326T	Western Blot	1:1000

Supplementary Table 4. The primers for RT-qPCR.

Primer name	Sequence
miR-4663--F	5′-AGCTGAGCTCCATGGACGTGCAGT-3′
miR-6084-F	5′-TTCCGCCAGTCGGTGGCCGG-3′
miR-6759-5p-F	5′-TTGTGGGTGGGCAGAAGTCTGT-3′
miR-6887-5p-F	5′-TGGGGGGACAGATGGAGAGGACA-3′
ANGPTL-F	5′-GTCCACCGACCTCCCGTTA-3′
ANGPTL-R	5′-CCTCATGGTCTAGGTGCTTGT-3′
SP1-F	5′-TGGCAGCAGTACCAATGGC-3′
SP1-R	5′-CCAGGTAGTCCTGTCAGAACTT-3′
HIF1A-F	5′- GAACGTCGAAAAGAAAAGTCTCG-3′
HIF1A-R	5′- CCTTATCAAGATGCGAACTCACA-3′
GAPDH-F	5′-AATGGGCAGCCGTTAGGAAA-3′
GAPDH-R	5′-GCGCCCAATACGACCAAATC-3′
U6-F	5′-CTCGCTTCGGCAGCACA-3′
U6-R	5′-AACGCTTCACGAATTTGCGT-3′

Supplementary Table 5. The primers for ChIP RT-qPCR

Primer name	Sequence
miR-6084-promoter F	5′- CGTGGGTCCAAAGTGCAAAG -3′
miR-6084-promoter R	5′- AGCAGCAGCGCTCGAC -3′

Supplementary Table 6. The baseline characters of patients included.

	CRC patients without distant metastasis(n=10)	CRC patients with liver metastasis (n=10)	P
Gender，male/female	7/3	5/5	0.625
Age，years	59.0 ± 4.4	59.7 ± 3.6	0.903
BMI, Kg/m2	22.3 ± 3.0	22.2 ± 3.1	0.980
AFP, ng/mL	3.4 ± 2.1	2.6 ± 0.8	0.268
CEA, ng/mL	5.7 ± 5.5	18.4 ± 27.6	0.171
CA199, U/mL	173.1 ± 354.6	12.0 ± 9.0	0.168
CA125, U/mL	72.8 ± 177.0	17.5 ± 13.1	0.337
cT			0.638
2	1	1	
3	5	3	
4	4	6	
cN			0.069
0	5	1	
1	1	5	
2	4	4	

BMI, Body mass index；AFP，Alpha-fetoprotein；CEA, carcinoembryonic antigen; CA199, carbohydrate antigen 199；CA125，carbohydrate antigen 125；Quantitative data is represented by mean ± standard deviation, while categorical data is represented by number of examples
