# Supplementary material for: MicroRNA-6084 orchestrates angiogenesis and liver metastasis in colorectal cancer via extracellular vesicles
Source: JCI Insight. 2025 Jun 10;10(14):e189503. doi: 10.1172/jci.insight.189503 (PMC12288965; doi:10.1172/jci.insight.189503)
Supplement: Unedited blot and gel images [file jciinsight-10-189503-s042.pdf]

MicroRNA-6084 orchestrates angiogenesis and liver metastasis in colorectal cancer via extracellular vesicles

Unedited images for all blots and gels in the manuscript associated with Figures and Supplementary Figures

Full unedited gel for Figure 3E

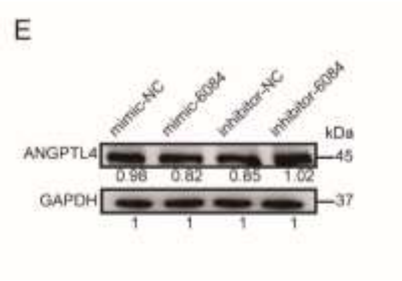

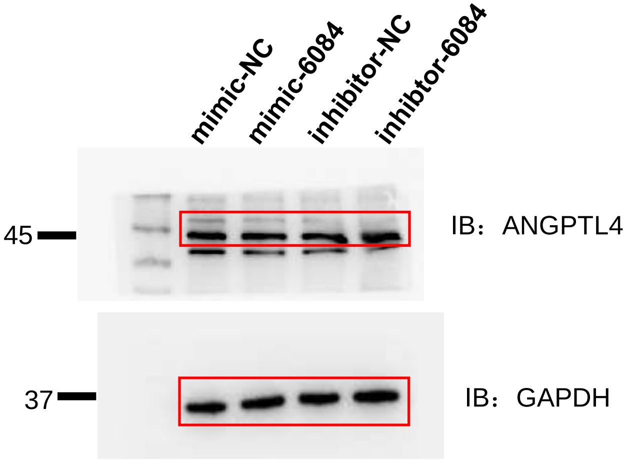

The blots come from the same batch of sample

Full unedited gel for Figure 4G

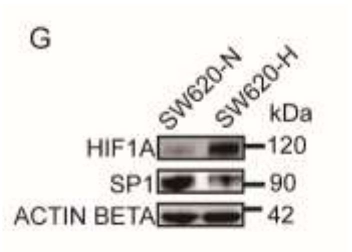

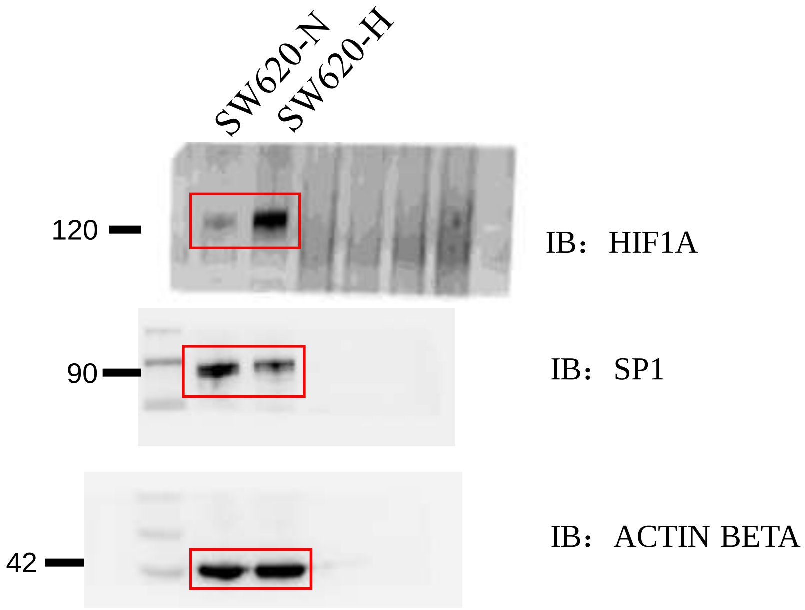

The blots come from the same batch of sample

Full unedited gel for Figure 4H

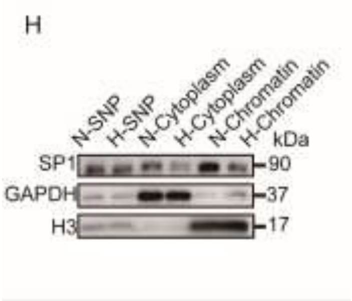

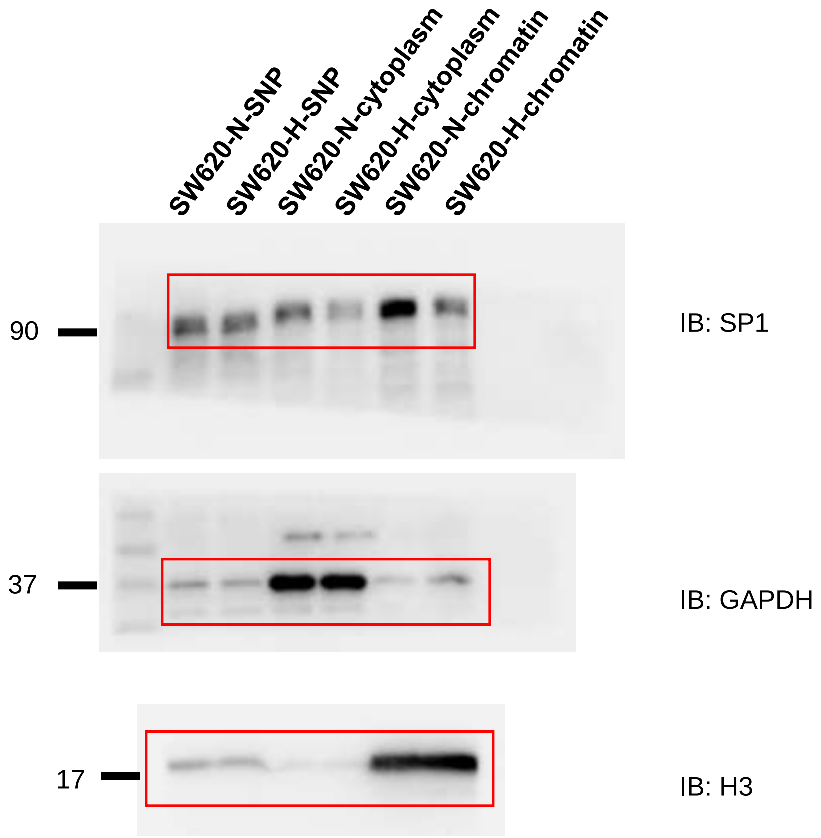

The blots come from the same batch of sample

Full unedited gel for Figure 4I

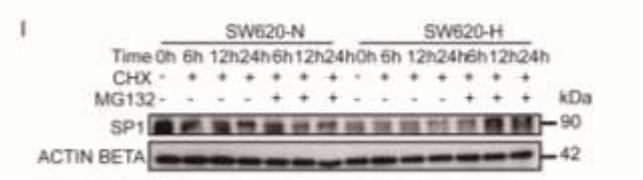

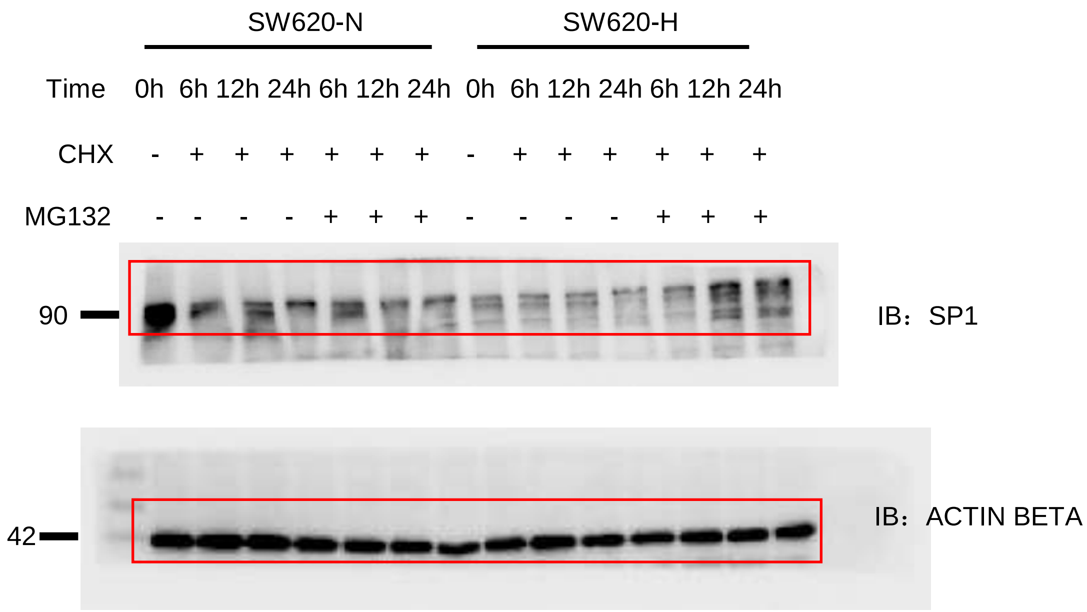

The blots come from the same batch of sample

Full unedited gel for Figure 4J

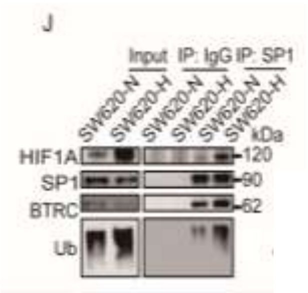

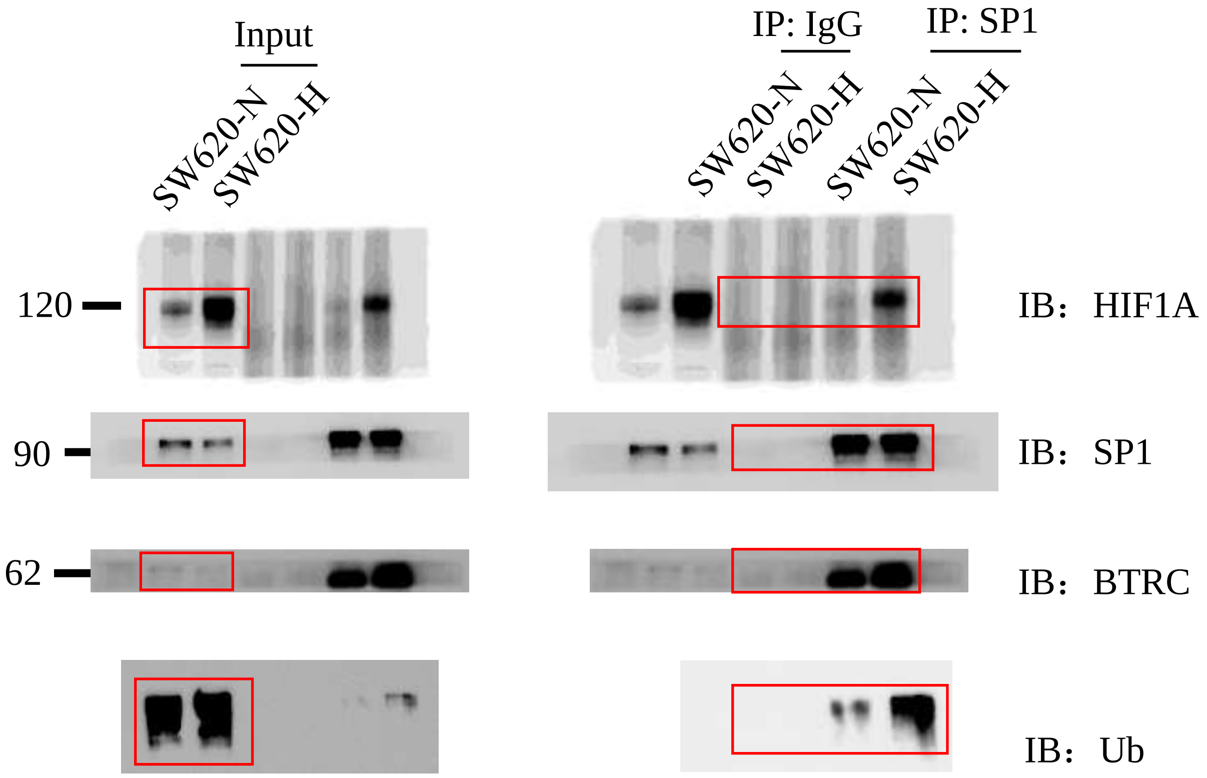

(This image shows the cut off of four holes on the right side of the left image and re-exposed)

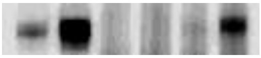

The blots come from the same batch of sample

Full unedited gel for Figure 4K

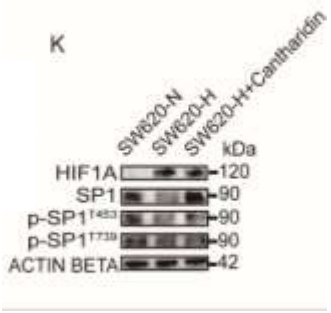

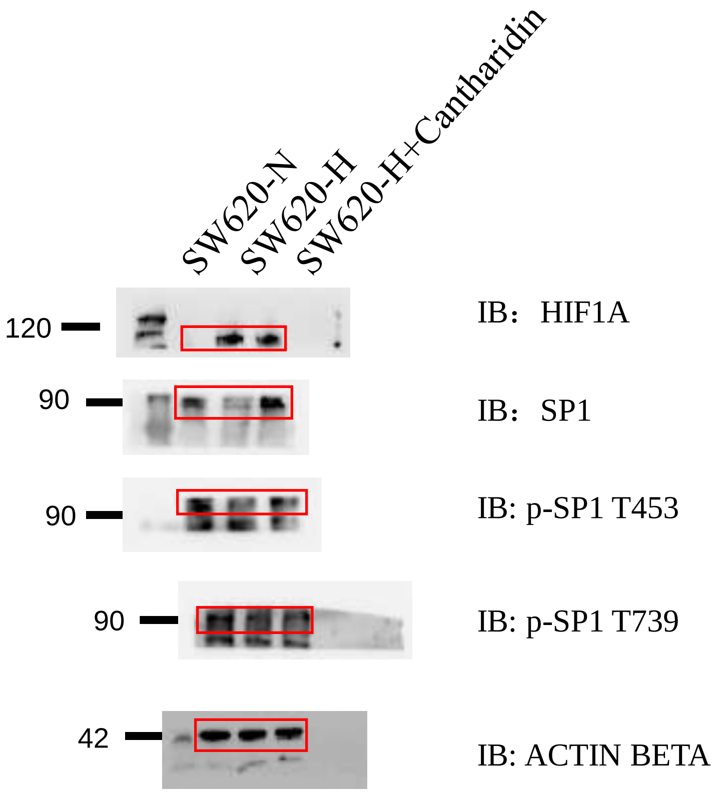

The blots come from the same batch of sample

Full unedited gel for Supplementary Figure 1A

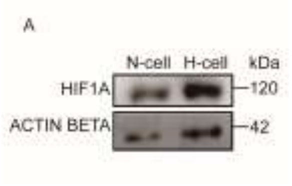

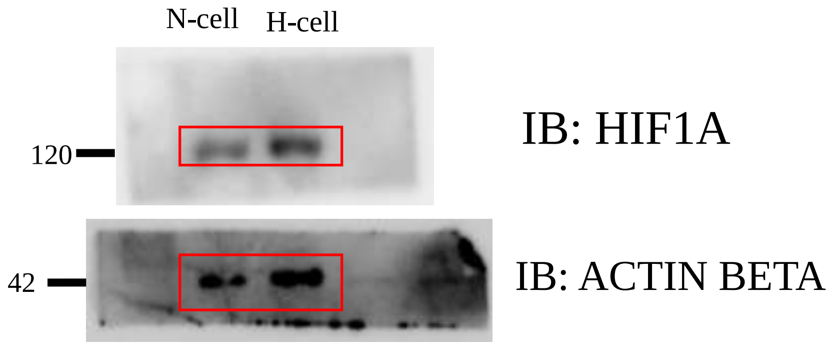

The blots come from the same batch of sample

Full unedited gel for Supplementary Figure 1D

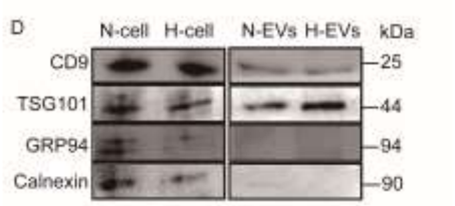

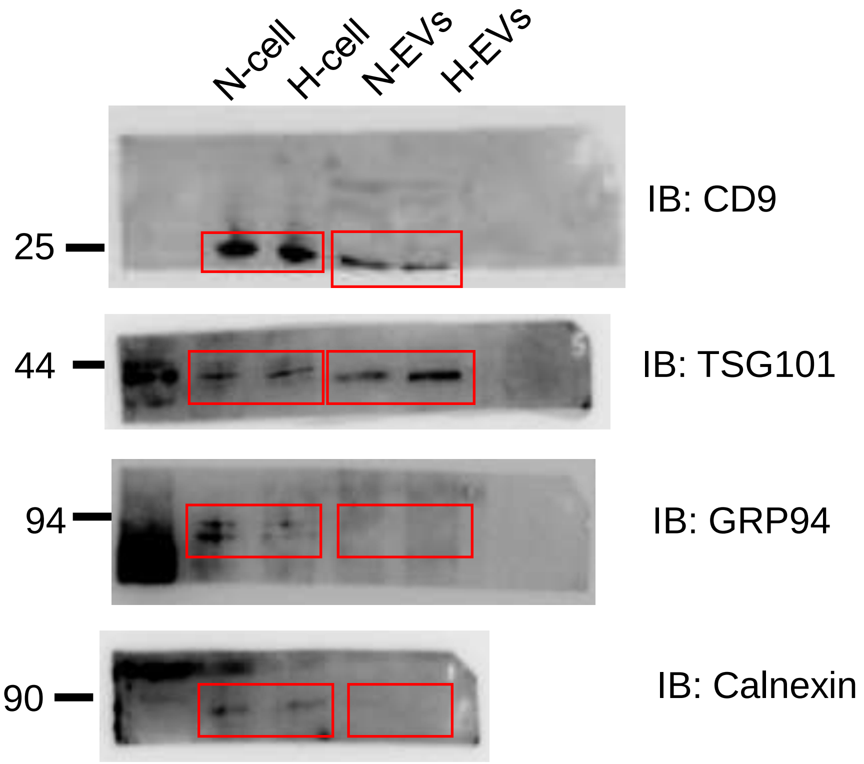

The blots come from the same batch of sample

Full unedited gel for Supplementary Figure 4B

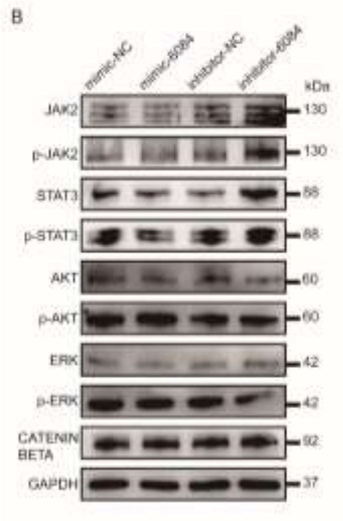

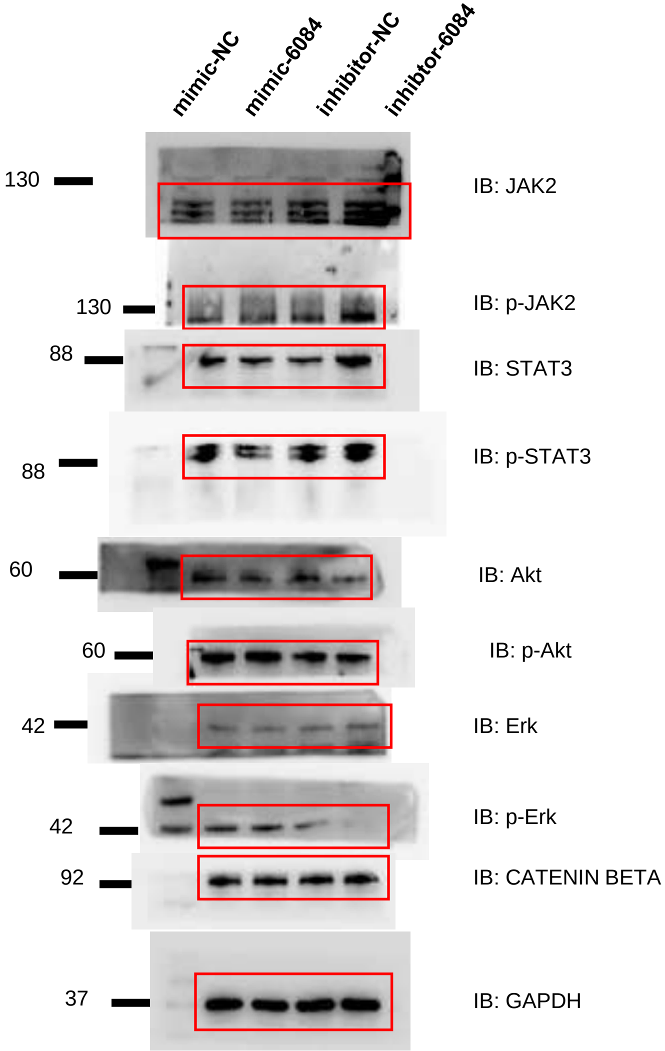

The blots come from the same batch of sample

Full unedited gel for Supplementary Figure 4C

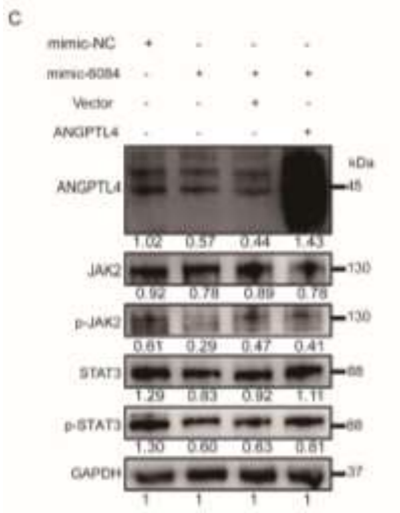

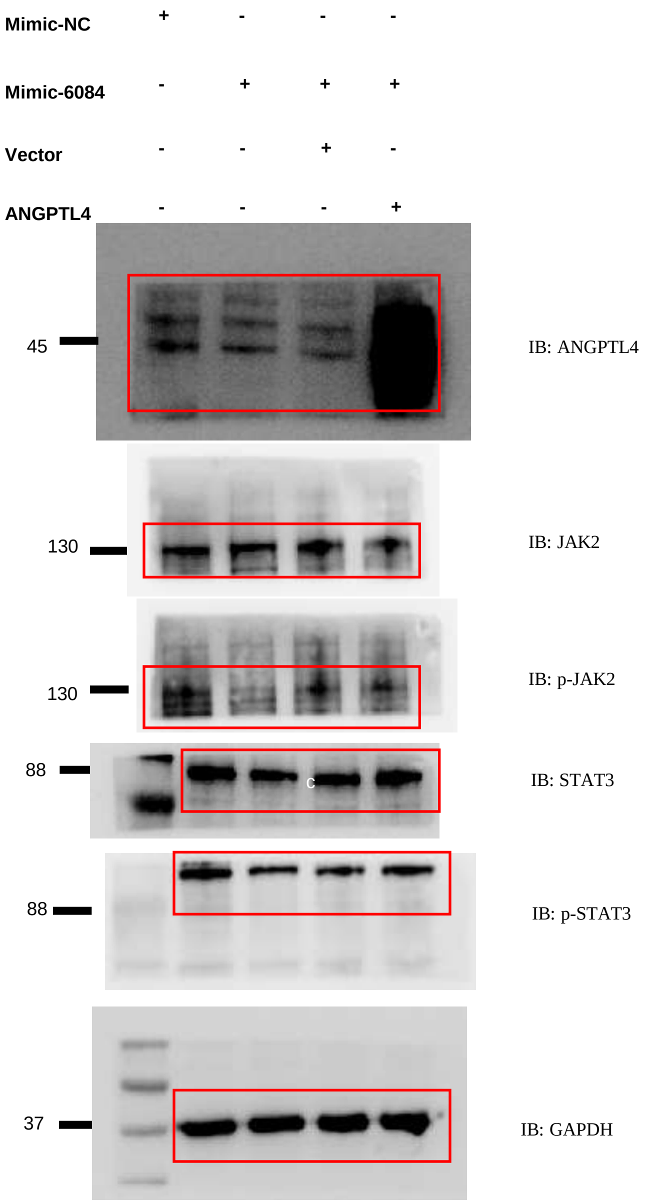

The blots come from the same batch of sample

Full unedited gel for Supplementary Figure 4F

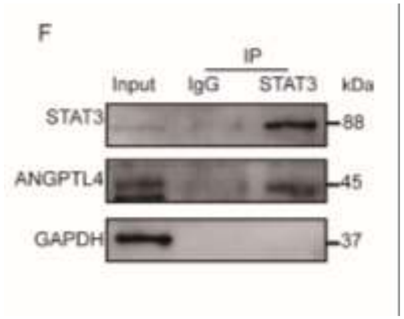

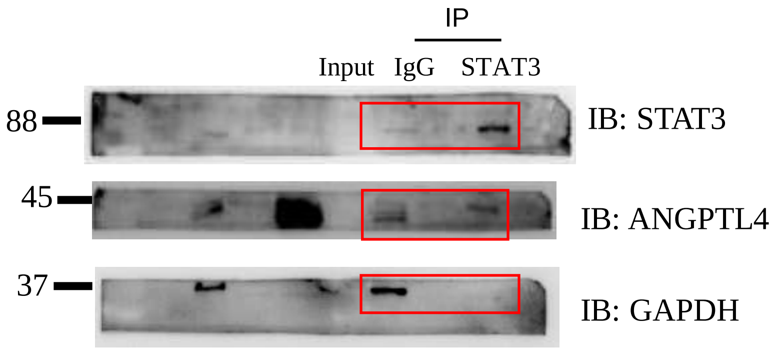

The blots come from the same batch of sample

Full unedited gel for Supplementary Figure 5D

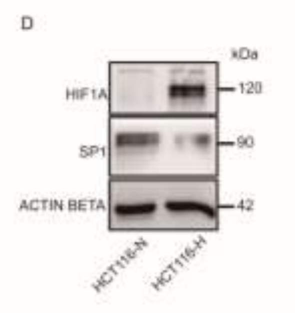

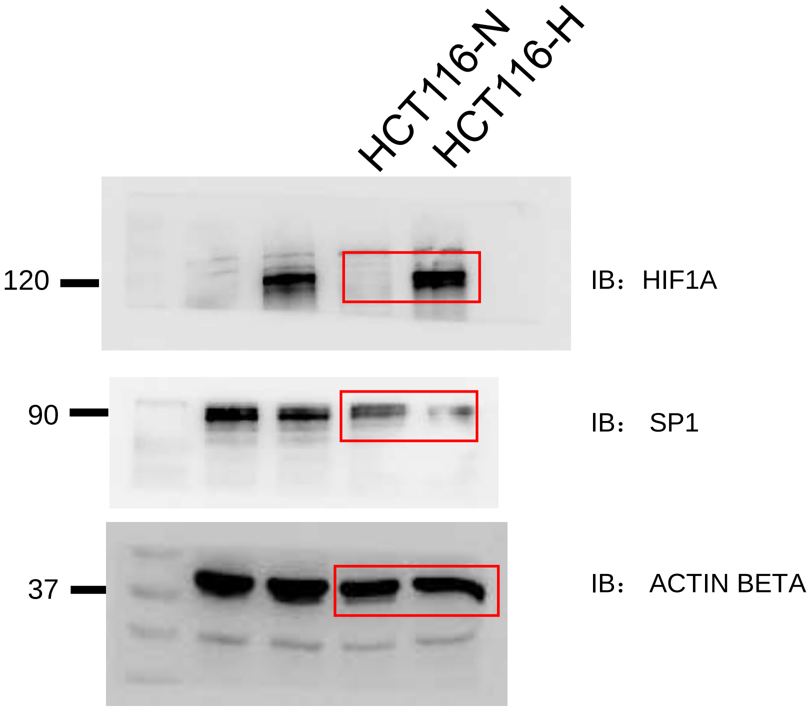

The blots come from the same batch of sample

Full unedited gel for Supplementary Figure 6A

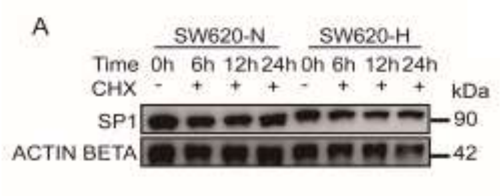

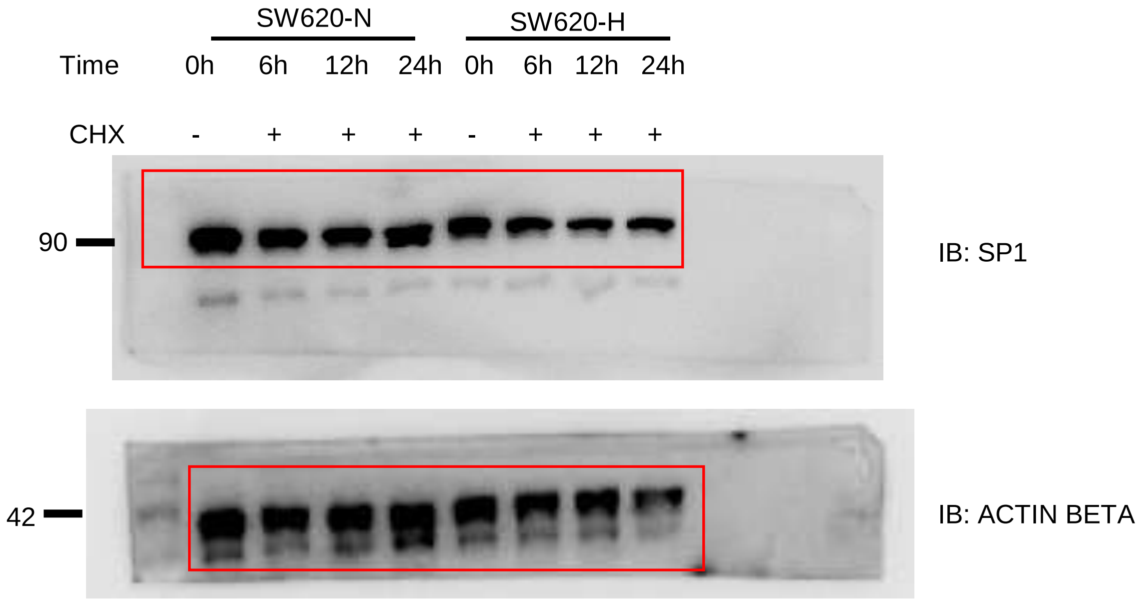

The blots come from the same batch of sample

Full unedited gel for Supplementary Figure 6C

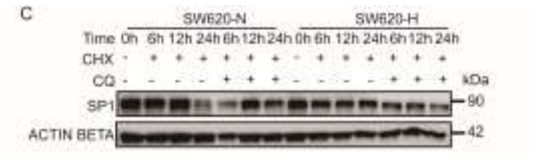

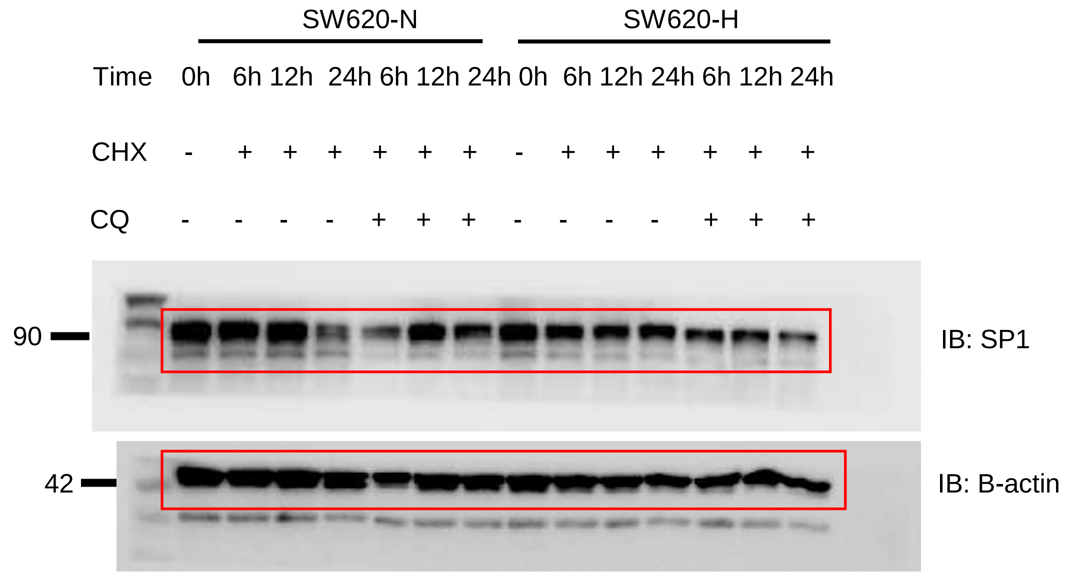

The blots come from the same batch of sample

Full unedited gel for Supplementary Figure 7B

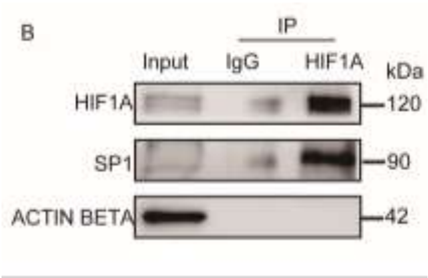

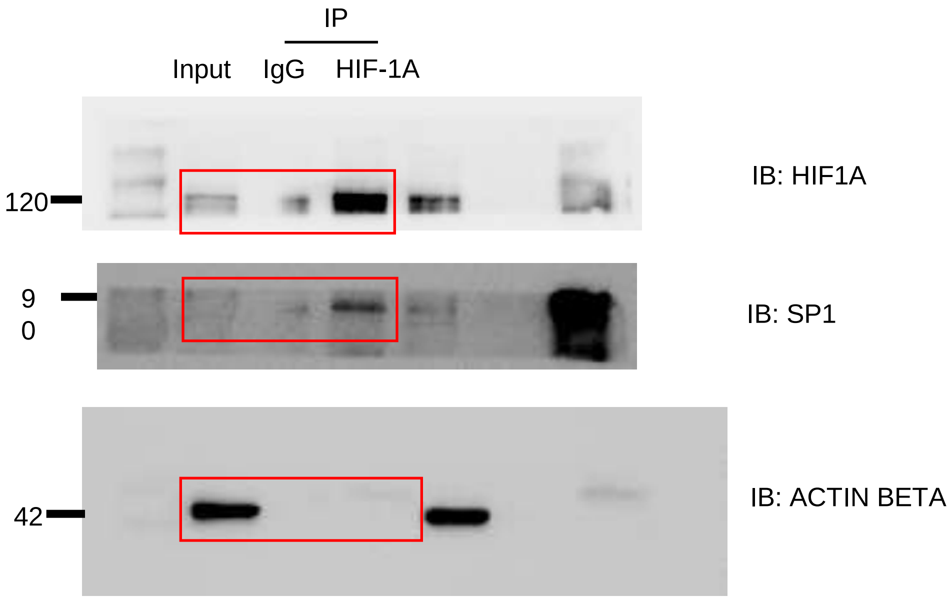

The blots come from the same batch of sample

Full unedited gel for Supplementary Figure 7C

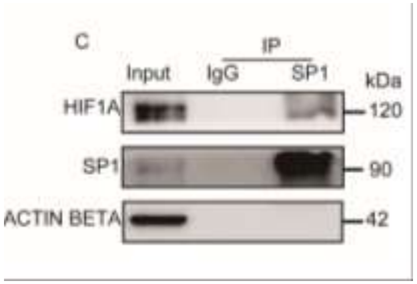

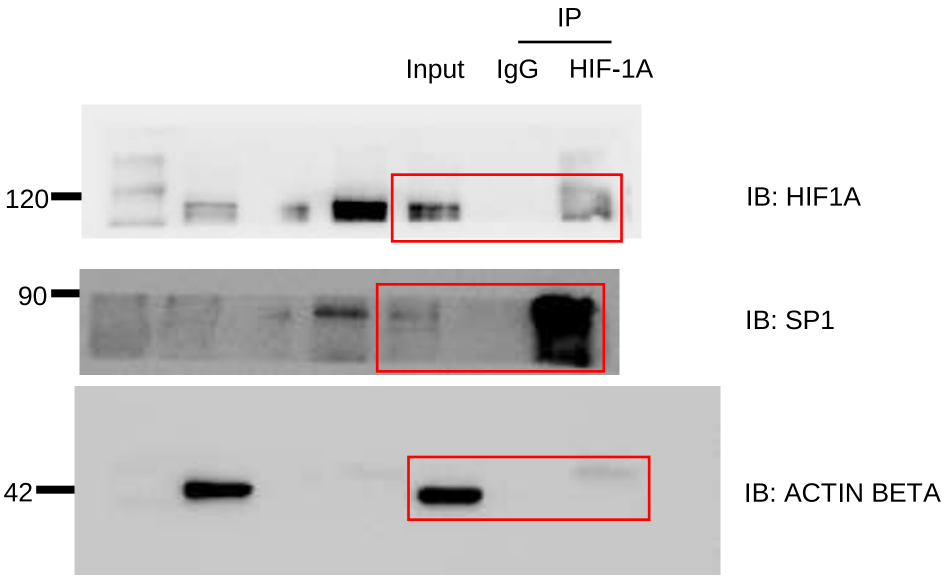

The blots come from the same batch of sample

Full unedited gel for Supplementary Figure 10C

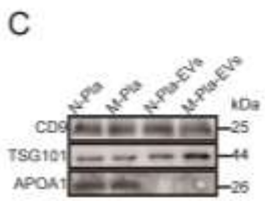

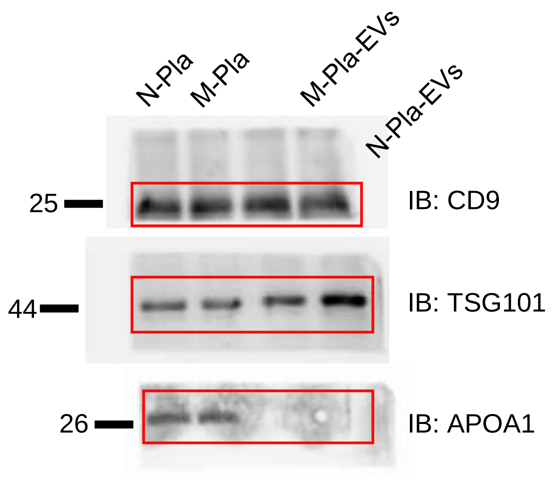

The blots come from the same batch of sample
